# A single layer artificial neural network type architecture with molecular engineered bacteria for reversible and irreversible computing†

**DOI:** 10.1039/d1sc01505b

**Published:** 2021-11-09

**Authors:** Kathakali Sarkar, Deepro Bonnerjee, Rajkamal Srivastava, Sangram Bagh

**Affiliations:** Biophysics and Structural Genomics Division, Saha Institute of Nuclear Physics, Homi Bhabha National Institute (HBNI) Block A/F, Sector-I, Bidhannagar Kolkata 700064 India sangram.bagh@saha.ac.in

## Abstract

Here, we adapted the basic concept of artificial neural networks (ANNs) and experimentally demonstrate a broadly applicable single layer ANN type architecture with molecular engineered bacteria to perform complex irreversible computing like multiplexing, de-multiplexing, encoding, decoding, majority functions, and reversible computing like Feynman and Fredkin gates. The encoder and majority functions and reversible computing were experimentally implemented within living cells for the first time. We created cellular devices, which worked as artificial neuro-synapses in bacteria, where input chemical signals were linearly combined and processed through a non-linear activation function to produce fluorescent protein outputs. To create such cellular devices, we established a set of rules by correlating truth tables, mathematical equations of ANNs, and cellular device design, which unlike cellular computing, does not require a circuit diagram and the equation directly correlates the design of the cellular device. To our knowledge this is the first adaptation of ANN type architecture with engineered cells. This work may have significance in establishing a new platform for cellular computing, reversible computing and in transforming living cells as ANN-enabled hardware.

## Abstract 2

We created artificial neural network type architecture with engineered bacteria to perform reversible and irreversible computation. This may work as new computing system for performing complex cellular computation.

## Graphical abstract

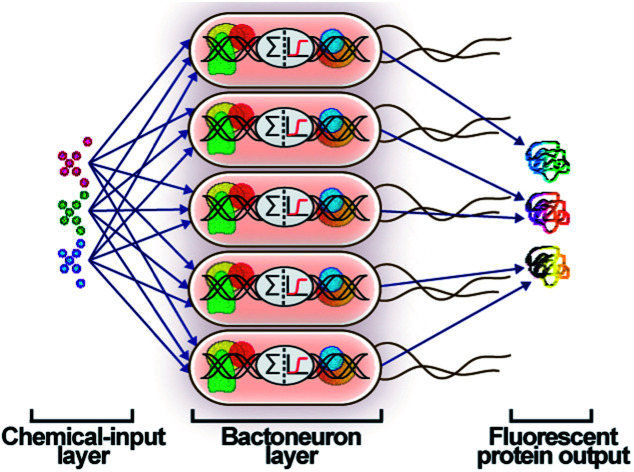

## Introduction

An Artificial Neural Network (ANN), partly inspired by the biological neurons in the brain, is a computing system where a set of nodes, called artificial neurons is connected with appropriate mathematical equations within a network and is able to map complex nonlinear systems.^1^ Though ANN computing has mostly been performed through software,^1–3^ hardware implementation of ANN through neuro-synapse type architectures^4–7^ has also been realized through various physical mechanisms in inorganic material based chips,^4–12^ photonics,^11^ and spintronics.^12^ ANN hardware has also been realized by exploiting chemical reactions using a copper catalyzed autocatalytic azide–alkyne cycloaddition^13^ and biochemical reactions using *in vitro* DNA computation.^14,15^ Furthermore, this inspires adaptation of the basic ANN type architecture with living cells to create complex artificial computing functions by engineering interactions at the molecular level.

The advent of synthetic biology has allowed implementation of engineering principles in the molecular and cellular biology regime, where many genetically encoded cellular devices, also called synthetic genetic circuits, have been created to carry out various computational operations.^16–18^ Synthetic genetic circuits perform logical operations by engineered transcriptional and translational machinery. Such systems may have applications in quantitative and mechanistic understanding of various natural cellular phenomena from the bottom-up,^19–21^ programmed therapeutics,^22,23^ biocomputation,^24,25^ and smart living materials.^26^ One of the major approaches in synthetic biology is adapting electronic circuit principles to create complex computing functions, where synthetic genetic logic gates^27–30^ were layered analogously to the electronic circuit design to create integrated genetic logic circuits and devices.^31–35^ Electronic analogous devices have been created in bacterial and mammalian cells. Some of the examples include basic logic gates,^27–30^ half adders,^31^ counters,^32^ DeMux and Mux^33^ in bacteria, single bit full adders^34^ and an analog to digital converter^35^ in mammalian cells. These circuits were either realized in a single cell^27–29,32^ or distributed among multiple cells.^30,33–35^ Such system development remains difficult, is not properly scalable and is not streamlined.^18,30,36^

Here we demonstrate a different computing system to create complex computing functions in living cells by adapting the basic concept of ANN. A feed forward ANN may approximate a wide variety of functions.^1^ Thus, it may be possible to create complex computing functions using an ANN type architecture with engineered bacteria. In this study, we experimentally created a broadly applicable single layer ANN type framework using engineered cellular devices in living bacteria for performing complex reversible and irreversible computation. Here, the cellular devices inside bacteria work as artificial neuro-synapses and we herein refer to such devices as ‘bactoneuron’ (BNeu). The cellular devices linearly combine the chemical inputs and transform them nonlinearly to a fluorescent protein output. Here, we established a set of general rules to map the complete ANN architecture and to derive unit bactoneurons directly from the functional truth table of a complex computing function without using its electronic integrated circuit design. Furthermore, we developed straightforward and universal molecular design rules directly from the mathematical nature of the activation function, which connect the molecular design of an individual cellular device and the sign of weights in an activation function of a bactoneuron in a one-to-one fashion.

We experimentally demonstrated that single-layer neural network type architectures that stemmed from those bactoneurons were general, flexible and perform complex irreversible computation through a 2-to-4 decoder, a 4-to-2-priority encoder, a majority function, a 1-to-2 de-multiplexer, and a 2-to-1 multiplexer and reversible logic mapping through Feynman and Fredkin gates. To our knowledge, the encoder and majority function have not been demonstrated and reversible computing has never been explored in living biological systems.

The bactoneuron ANN approach consists of a set of new well-defined rules to create a wide variety of complex computing functions. The ANN type architectures with engineered bacteria have several advantages over conventional integrated genetic circuit design: (i) a single layer ANN architecture does not require layering of logic gates, (ii) unlike the mathematical modeling of gene circuits in the conventional method, which requires various mathematical equations for various genetic logic gates, the ANN framework required only a single type of mathematical equation, (iii) the molecular design of the cellular device can directly be obtained from the mathematical equation of an activation function. Thus, ANN with bactoneurons may serve as a new, complementary and streamlined platform for creating reversible and irreversible computation with bacteria.

## Results and discussion

### Principles of mapping the functional truth table to the single layer molecular engineered bacterial ANN

**Fig. 1:**
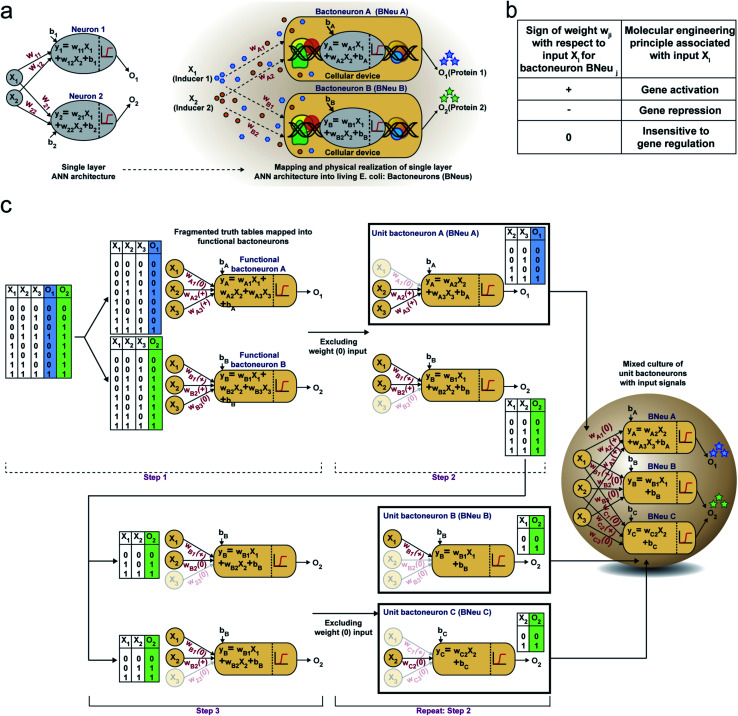
Single layer artificial neural network (ANN) type architecture with engineered bacteria and schematic design rules of building complex computing functions. (a) Schematic representation of an ideal single layer ANN with two weighted inputs (*X*_1_ and *X*_2_) and outputs (*O*_1_ and *O*_2_) along with their corresponding weights (*w*_*i*_), the biases (*b*_*i*_) and summation function (*y*_*i*_). This abstract ANN is mapped with the proposed artificial bacterial neurons (bactoneurons or BNeus). (b) Relationship between signs of weights in the activation function and molecular engineering principles for a bactoneuron associated with an input. (c) Making of a bacteria-based single layer ANN type architecture from the truth table of a given function.

First, we hypothesized that an abstract ANN model can be mapped into an engineered cellular model (Fig. 1a), where engineered cellular devices inside bacterial cell work as artificial neuro-synapses (bactoneurons). The bactoneurons combined the inputs in the form of environmental chemical inputs and those inputs were processed by synthetic genetic circuits, which work through engineered transcriptional regulation to execute appropriate log-sigmoid activation functions (eqn (1)). We call these synthetic genetic circuits ‘cellular devices’. Eqn (1) is a conventional activation function for characterization of an artificial neuro-synapse in ANN^1^ with two inputs and can be applied to a wide range of functional behaviors, based on its sign and magnitudes of the weight and bias terms.

In this equation, each bactoneuron has two weight values of varying signs and magnitude corresponding to its two chemical inducer inputs (*X*_1_ and *X*_2_) and a bias with its value in accordance with the functional response of the neuron. For a given bactoneuron *j*,1
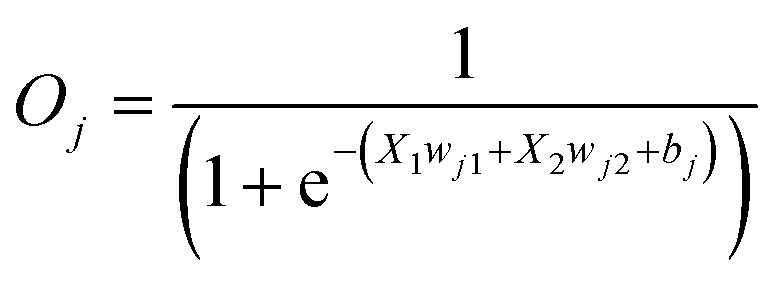
where *O*_*j*_ is the output from neuron *j*, *X*_1_ and *X*_2_ represent two input inducer concentrations, *w*_*j*1_ represents the weight of input *X*_1_ for the neuron *j*, *w*_*j*2_ represents the weight of input *X*_2_ for the neuron *j*, *b*_*j*_ represents the bias for the neuron *j*.

Eqn (1) suggests that if *w*_*j*1_ is positive, output *O*_*i*_ would increase with *X*_1_. In terms of cellular devices, it is similar to a molecular activation (Fig. 1b). Similarly, a negative weight would suggest a repression and ‘zero’ weight suggests the insensitivity of the input with the output (Fig. 1b). This simple correlation between the sign of a weight, *w*_*ji*_ within an activation function of a ‘unit’ bactoneuron and molecular engineering principles, guided the physical design of the cellular device.

Next, we devised a way to map a complex computing function through a single layer ANN type architecture directly from its functional truth table, without considering its hierarchical electronic design principle (Fig. 1c). We built a set of rules to derive bactoneurons from the functional truth table by dividing the bigger truth tables into smaller ones and to connect them with the bactoneuron design (Fig. 1c). We demonstrated this process considering a random functional truth table (Fig. 1c). First, we considered a single output within a functional truth table and then looked at its relationship with all the input combinations. We grouped those input combinations in the form of smaller truth tables, in such a way that each input corresponding to that particular output possessed a weight with only one type of sign (+, − or 0) (step 1). Such individual bactoneurons in this step were named ‘functional bactoneurons’, which in appropriate ANN combinations would give rise to the actual function. Next, we ignored the weight(s) with ‘zero’ values, if any, from functional bactoneurons and mapped them back with smaller truth tables (step 2). Furthermore, we looked at the output of the smaller truth table from step 2. If the output value 1 (true) appeared only once in the smaller truth tables, we defined them as ‘unit bactoneurons.’ Otherwise, we kept dividing the truth table (step 3) until the above condition appeared. This way we identified the unit bactoneurons, which is the smallest unit required to be constructed as a cellular device. Once those unit bactoneurons are combined appropriately, they would operate as functional bactoneurons. Thus, when the unit bactoneurons were assembled according to the ANN structure, the actual function was physically realized (Fig. 1c).

**Fig. 2:**
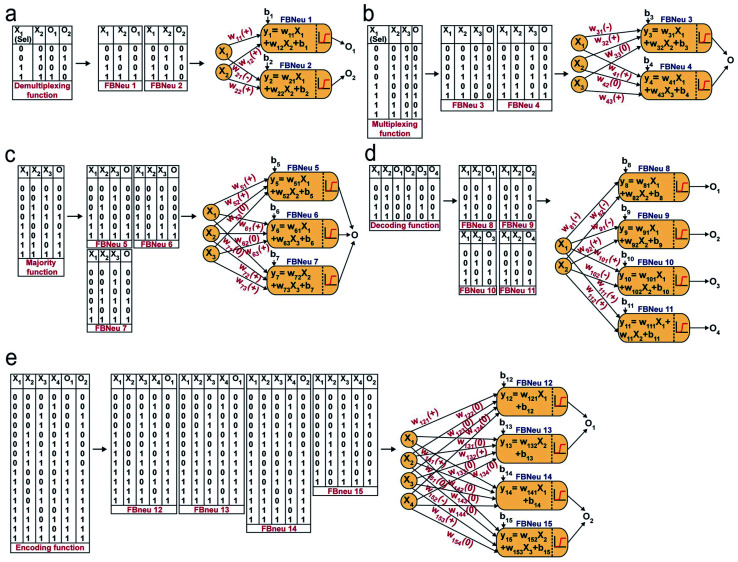
Derivation of functional bactoneurons and abstraction of single layer ANN type architectures from functional truth tables of complex computing functions. (a) 1-to-2 de-multiplexer, (b) 2-to-1 multiplexer, (c) 3-input majority function, (d) 2-to-4 decoder and (e) 4-to-2 priority encoder.

Next, we chose a range of computing functions with varying complexities (Fig. 2) and derived their functional bactoneurons (Fig. 2) from their functional truth table following the principle stated above (Fig. 1c). The chosen functions included 1-to-2 demultiplexer^37^ (Fig. 2a), 2-to-1 multiplexer^37^ (Fig. 2b), majority functions^38^ (Fig. 2c), 2-to-4 decoder^37^ (Fig. 2d), and 4-to-2 priority encoder^37^ (Fig. 2e). A de-multiplexer performs as an output selector where it takes input from just one source and the logical state(s) of selector line(s) direct(s) to select only one among multiple output channels to process the signal and interpret it. The multiplexer performs the complementary function where the logical state(s) of selector line(s) directs which input is to be received for generating an output. A majority function suggests that in a ternary system if more than 50% of the inputs are true then the output is true, otherwise false. A N:2N decoder converts N bit binary-coded inputs into 2N coded outputs in a one-to-one mapping fashion and an encoder encodes input signals to fewer bits and transforms them into encoded outputs. The details of deriving functional and unit bactoneurons from the truth tables of all those functions without considering its integrated circuit design are shown in Fig. 2 and ESI Fig. S1.† The specific activation function equations for all functional bactoneurons are shown in Table S1.†

### Design and construction of cellular devices and unit bactoneurons

Next, we developed cellular devices to construct the unit bactoneurons (Table S1†) in our chassis organism, *E. coli* DH5αZ1.^39^ The cellular devices are engineered molecular networks incorporated in plasmid vectors, which are 25 nm circular DNAs replicate inside the bacteria.^40^ In our design, the abstract inputs (*X*_*i*_) were replaced by extracellular chemical inducers like isopropyl β-d-1 thiogalactopyranoside (IPTG), anhydrotetracycline (aTc), *N*-acyl homoserine lactone (AHL), and arabinose. The abstract outputs (*O*_*i*_) were changed to fluorescent proteins like EGFP, mKO2, E2 Crimson, mTFP1, and mVenus as appropriate. The device design of the unit bactoneurons were based on the molecular engineering principle we stated in Fig. 1b.

**Fig. 3:**
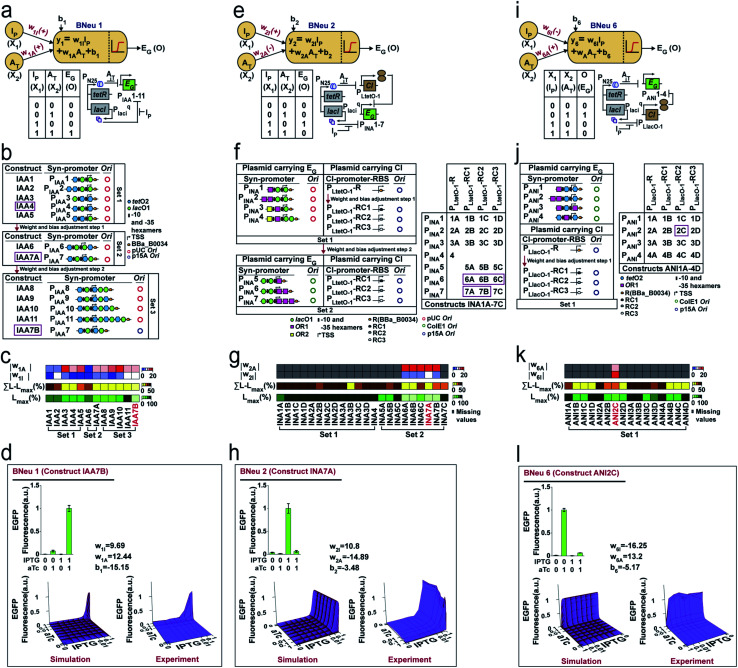
Design, experimental characterization, and weight and bias adjustments of cellular devices for unit bactoneurons BNeu 1, BNeu 2 and BNeu 6. (a) Neural architecture of the bactoneuron BNeu 1 where *w*_1I_ and w_1A_ are weights of inputs IPTG (I_P_: *X*_1_) and aTc (A_T_: *X*_2_), and *b*_1_ is the bias. Activation function generates output EGFP (E_G_: O). Truth table and biological circuit design of the BNeu 1 activation function. P_IAA_1–11 are synthetic promoters designed for BNeu 1 activation function and regulated by IPTG and aTc as these promoters contain binding cites for LacI and TetR proteins. (b) Schematic representation of weight and bias adjustment of BNeu 1 through constructs IAA1–11. IAA1–11 were generated in sets by changing the hybrid promoter design with varied number and positions of the operating sites for both TetR and LacI and by simultaneously changing the origin of replication (Ori) of the plasmids carrying those promoters. Promoter maps of P_IAA_1–11 are also shown. Positions of −10 and −35 hexamers, transcription start site, ribosome binding sites (RBS) and LacI & TetR binding sites are depicted in individual promoter maps. Weights and bias associated with IPTG and aTc of the BNeu 1 were adjusted through a two-step modification of molecular interactions. Constructs corresponding to the selected promoter from each set are shown in the magenta box. (c) Heatmaps showing percentage highest leakage (*L*_max_(%)), percentage sum of leakage excluding the highest leakage (∑*L* − *L*_max_(%)), modulus of weight associated with IPTG (|*w*_1I_|) and modulus of weight associated with aTc (|*w*_1A_|) for each out of 12 constructs (constructs IAA1–11). Construct IAA7B (coloured in red) was selected as the best performer. (d) Expression characterization, simulated behavior (3D plot), and experimental validation of unit bactoneuron BNeu 1 carrying construct IAA7B are shown. All of these experimental data were collected after 10 h induction followed by resuspension and 6 h induction. (e) Neural architecture, truth table and biological circuit design of the bactoneuron BNeu 2 activation function. (f) Weight and bias adjustment of BNeu 2 through constructs INA1A-7C, which are a two-plasmid system. Here, we re-engineered the synthetic promoter of BNeu 1 and replaced the aTc gene-activation function (+*w*) with aTc gene repression function (−*w*), such that in the presence of aTc, transcription from the promoter gets turned off. In this promoter we introduced an operating site for λ repressor CI proteins and the amount of CI was under the control of an aTc-inducible promoter. Promoter maps, *Ori*, RBS, and its various combinations in constructs are shown. (g) Heat maps of weight, leakage and hence bias adjustment for BNeu 2. (h) Characterization, simulation and validation of BNeu 2 (INA1A-7C). (i) Neural architecture, truth table and biological design of BNeu 6. (j) Details of the constructs (ANI1A-4D) for weight and bias adjustment of BNeu 6 and (k) corresponding heat maps are also shown. Construct ANI2C was chosen as the best performing construct. (l) Characterization, simulation and validation of BNeu 6 construct ANI2C.

We started with the construction and characterization of unit bactoneuron BNeu 1 (Fig. 3a–d), where both the weights in the activation function are positive with respect to the inputs (*X*_1_ and *X*_2_). In BNeu 1, two inducer chemicals IPTG and aTc were used as the inputs *X*_1_ and *X*_2_ respectively while enhanced green fluorescence protein (EGFP) was used as the output *O*_1_. Previously, IPTG and aTc induced synthetic promoters P_LlacO-1_ and P_LtetO-1_, respectively, in *E. coli* were shown demonstrating nonlinear behavior between the inducer concentration and reporter protein expression.^39^ Therefore, to design our hybrid synthetic promoters for BNeu 1 we adapted the basic promoter design of those promoters. The cellular device (Fig. 3a) for BNeu 1 consists of a synthetic hybrid promoter, which combines the chemical signals aTc and IPTG and processes them through a log-sigmoid function (eqn (1)) and according to the principle, both aTc and IPTG should work as activators for the system. TetR and LacI, two transcription factors, which are constitutively and endogenously expressed in *E. coli* DH5αZ1, bind the hybrid promoter thereby hindering it from expressing EGFP.^39^ Both IPTG and aTc bind with LacI and TetR, respectively, and change their conformation such that they cannot bind to the promoter anymore, and the promoter is free to recruit RNA polymerase for EGFP expression.

To start with random weights and biases, as in any ANN design,^1^ we constructed and characterized an initial set (Set 1) of molecular constructs (IAA1-5) (Fig. 3b and Table S2†), containing synthetic promoters P_IAA_1–P_IAA_5 respectively (Table S3†). For this, we measured the EGFP expression at various combinations of ‘zero’ and ‘saturated’ concentration of IPTG and aTc (Fig. S2a†) and performed dose responses (Fig. S2b†) of EGFP expression as a function of IPTG/aTc, by varying the concentration of one chemical, while keeping the other at saturated concentration. The dose–response behaviors were fitted to a modified form of eqn (1) (eqn (2), ESI note 1†). The fitting parameters gave the values of weights for each input and biases (Table S4†). This starting set (Set 1) showed high leakage and lower weight values (Fig. 3c, S2a, S2b, Tables S4 and S5†). Leakage is defined as the basal level expression of the reporter fluorescent proteins under the input conditions, where output expression should be zero.

Higher values for *w*_1I_ and *w*_1A_ would signify a sharper transition from the OFF to ON state and more negative bias might signify a reduced intercept in a dose–response curve, which could get reflected as less ‘leakage’, which was defined by the basal EGFP expression.

Guided by the least leakage and reasonably high weight values for both *w*_1I_ and *w*_1A_, we chose to construct IAA4 where the promoter P_IAA_4 served as a platform and adjusted the weights and bias by tweaking the molecular design and further creating new constructs (Fig. 3b and Tables S2 and S3†). We iteratively performed this adjustment process several times (Fig. 3c). Clearly, the weight ‘*w*’ of a bactoneuron was a strong function of the types and degree of molecular interactions, as evident from the fact that the weight of the initial bactoneuron was adjusted to a new one in each iteration. We found *w*_1I_ as the limiting weight as this had a lower value than *w*_1A_ (Fig. S2b and Table S4†). In addition, we focused on the highest leakage value (*L*_max_), and (∑*L* − *L*_max_) of each construct, where ∑*L* is the total leakage (Table S5†). Our goal was to reduce it. Constructs IAA4 and IAA5 from the first set carried similar weights but IAA5 showed significantly high ∑*L* − *L*_max_. Thus, IAA4 was chosen for further adjustment. Fig. 3c shows the adjustment of values for weights and leakage. The constructs for further adjustment in each step are boxed. Now, the construct IAA7A (for BNeu 1) from the second set of adjustment was taken for further weight adjustment either by engineering the promoter or by altering the relative numbers of the promoters per cell by changing the copy number of the plasmids (Fig. 3b). Construct IAA7B (Table S2†) had higher weight values and the least leakage with respect to construct IAA7A (Fig. 3c, Tables S4 and S5†). Others (IAA8–11) from the same iteration showed comparatively poor behavior. Such scenarios could be compared with overshooting of weight adjustment, as happens in the ANN.^1^ Thus, the cellular device IAA7B was selected as the unit bactoneuron BNeu 1. We performed a simulation and experimentally tested the behavior of BNeu 1 by simultaneously changing the concentration of the IPTG and aTc (Fig. 3d). The results show a close topological match with the simulation (Fig. 3d).

In an ANN framework, bias may determine the intercept.^1^ The leakage in the bactoneuron determines the intercept in the dose–response curves. A moderate correlation (*R*^2^ = 0.76) between the bias ‘*b*_1_’ and *L*_max_ was found (Fig. S3a†) within the experimental range for BNeu 1 construct IAA7B. However, in this case, adjustments of weight values were linked to that of the bias during iteration and it was difficult to distinguish the exact molecular reasoning. We performed a simulation by varying the bias but keeping the weights constant and it suggested that ‘bias’ manifested as leakage in BNeu 1 (Fig. S3b†). We performed similar simulations (Fig. S3†) for all unit bactoneurons (Table S1†) and the results suggested that the bias value in the bactoneuron indicated the leakage from the cellular devices within a parameter range.

We further illustrate the construction of two other bactoneurons namely BNeu 2 and BNeu 6, which showed positive weight for one inducer and negative for the other (Fig. 3e–h and i–l). The development of BNeu 1 indicated that *L*_max_ could be the first parameter to look for during molecular engineering. Therefore, for BNeu 2 and BNeu 6, we first checked if the fold change between the highest signal (output logic level “1”) and the highest leakage was more than 8 times (Table S5†). If it was so, we would proceed to adjust the weight values and linked-biases for optimal behavior of the corresponding unit bactoneurons either by engineering the *cis*–*trans* element interaction on the promoter, or by reducing the translation rate of CI *via* RBS designing (Table S6†) or by modulating the relative number of synthetic promoters per cell through modification of the plasmid copy number. We followed this processing pipeline to characterize, fit and adjust the weights and biases in iterations to get the unit bactoneurons BNeu 2 and BNeu 6 (Fig. 3e–h and i–l), executed by constructs INA7A and ANI2C respectively (Table S2†). The gene expression characterizations, dose–response and fitting for all constructs for BNeu 2 and BNeu 6 are shown in Fig. S2c, d and S2e, f† respectively. The design, gene expression characterizations, dose–response, fitting, simulation, and experimental validation of all other unit bactoneurons are shown in the Fig. S4.† In many cases the unit bactoneurons were equivalent to the functional bactoneurons which did not have any ‘0’ weight inducer input (insensitive to a certain inducer input). Therefore, for such bactoneurons, we experimentally validated the weight ‘zero’ characteristics with respect to the appropriate inputs (Fig. S5†). The details of the assumptions and process in designing and optimizing each of the bactoneurons are given in Table S7.†

### ANN created from molecular engineered bactoneurons generate complex computing functions

**Fig. 4:**
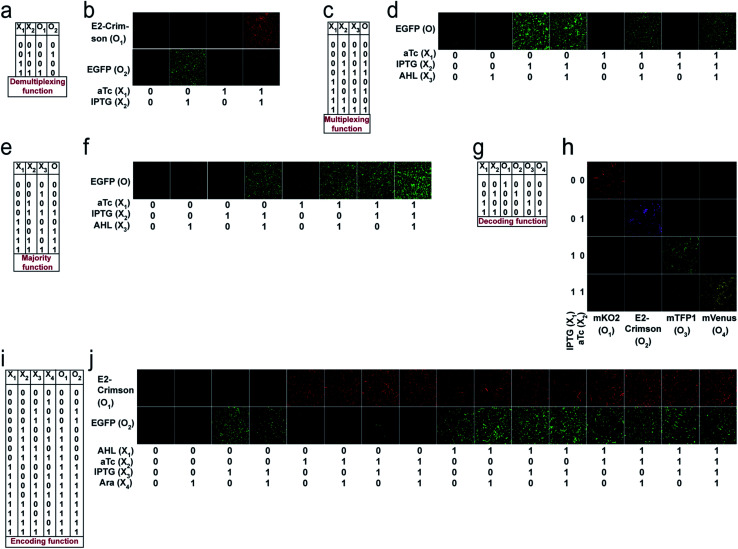
Experimental demonstration of complex computations with single layer ANN type architectures with molecular engineered bactoneurons. Truth tables of the (a) 1-to-2 de-multiplexer, (c) 2 to-1 multiplexer, (e) 3-input majority function, (g) 2-to-4 decoder and (i) 4-to-2 priority encoder are shown. Inputs and outputs are written as *X*_*i*_ (*i* = 1 to *n*) and *O*_*i*_ (*i* = 1 to *n*) respectively. Experimental behavior of the bacteria-based single layer ANN type architectures corresponding to the (b) 1-to-2 de multiplexer, (d) 2-to-1 multiplexer, (f) 3-input majority function, (h) 2-to-4 decoder and (j) 4-to-2 priority encoder, studied with a fluorescence microscope. Unit bactoneurons of a function were cultured in a mixed population and treated with all possible combinations of inputs. The resultant expressions of fluorescent proteins are represented by separate output channels.

For unit bactoneuron construction, we used EGFP as an output. We changed the EGFP with mKO2, E2-Crimson, mTFP1, and mVenus (Table S1†) as appropriate. Next, the unit bactoneurons were mixed, cocultured and exposed to various combinations of input chemicals following relevant ANN designs (Fig. 2). The experimental results are shown in Fig. 4 and S6† for the 1-to-2 de-multiplexer (Fig. 4a and b and S6a†), 2-to-4 multiplexer (Fig. 4c, d and S6b†), 3-input majority function (Fig. 4e, f and S6c†), 2-to-4 decoder (Fig. 4g, h and S6d†), and 4-to-2 priority encoder (Fig. 4i, j and S6e†). The results showed the expected truth table behavior (Fig. 4).

### Mapping logical reversibility with molecular engineered bactoneurons: Feynman and Fredkin gates

**Fig. 5:**
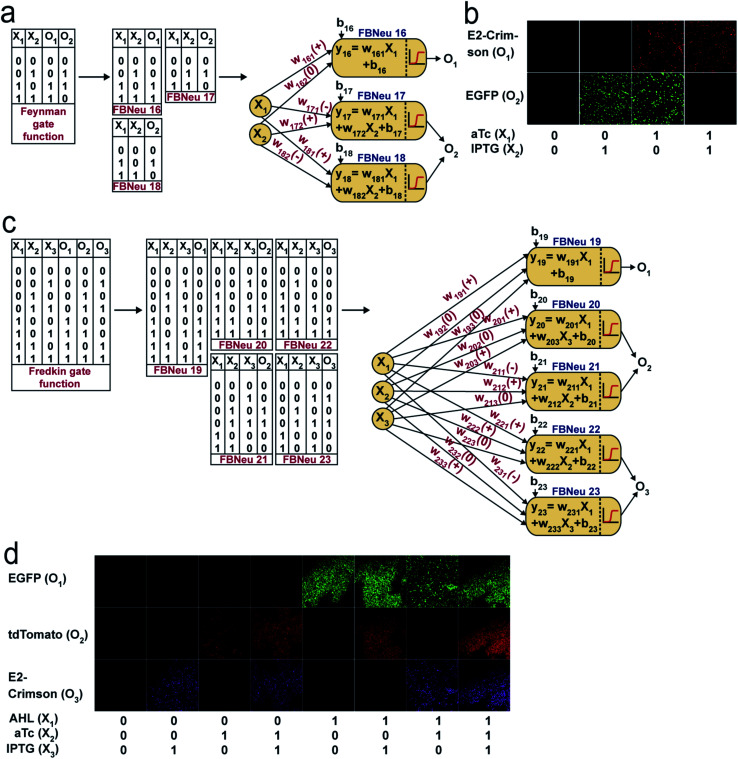
ANN architecture and experimental demonstration of reversible Feynman and Fredkin gates with molecular engineered bactoneurons. Derivation of functional bactoneurons from truth tables for (a) Feynman gate and (c) Fredkin gate. Experimental behavior of (b) Feynman gate and (d) Fredkin gate.

Reversible computing is the heart of quantum computing^41^ and it can map the previous state of the computation from the current state in a one-to-one basis.^41,42^ This is called logical reversibility, which was demonstrated by implementing logically reversible Fredkin and Toffoli gates through *in vitro* DNA computation.^43^ However, no logically reversible gate has been implemented in living cells. Although the thermodynamic reversibility of reversible computing, which gives the lowest energy cost in computation, is not possible in living systems, the potential of logical reversibility in biological systems is yet to be explored. We showed that the ANN with bactoneurons had the flexibility to create reversible computing and we demonstrated the universal reversible Feynman gate (Fig. 5a and b and S1f, S6f†) and Fredkin gate (Fig. 5c and d and S1g, S6g†), which may create any linear reversible logic gate. First we derived the functional and unit bactoneurons for Feynman (Fig. 5a and S1f, Table S1†) and Fredkin gates (Fig. 5c and S1g, Table S1†). The Feynman gate was represented by 3 unit bactoneurons (BNeu2, BNeu6, BNeu8), which we already developed. The ANN created from the corresponding functional bactoneurons (FBNeus 16–18) of these unit bactoneurons (Fig. 5a) showed a successful Feynman gate (Fig. 5b). The Fredkin gate was represented by 5 unit bactoneurons (BNeu 3, 4, 7, 9, 10), where BNeu 3, 4, and 7 were already developed, and BNeu 9 and BNeu 10 were created (Fig. S4p–u†). The ‘zero’ weights of the bactoneurons, where appropriate, are validated in Fig. S5.† The ANN, created from the corresponding functional bactoneurons (FBNeus 19–23) (Fig. 5c), showed a successful Fredkin gate (Fig. 5d).

## Discussion

Neurons are conventionally viewed as being the ultimate biological hardware, and these structures naturally form neural networks to perform extremely complex computational functions. Bacteria are not equipped to perform such complex tasks. Furthermore, in comparison to the millisecond speed of response of a biological neuronal network^44^ or nanosecond response time of ANN hardware,^45^ the transcriptional regulations in bacteria take tens of minutes to hours. However, bacteria are micron-size objects, which replicate fast and perform biological work by taking food from the environment, without the need for a battery. If a set of bacteria is programmed to perform complex computing tasks, they could be attractive chassis for creating microbiorobots, biohybrid-robots and cellular computers.^25,46,47^ The irreversible computing functions we developed (demultiplexer, multiplexer, decoder, encoder, majority function) were the major components in telecommunications, networking, and data transfer systems. Furthermore, the logical reversibility we demonstrated was a new class of computing functions created through biological cells. The ultimate goal of our work is to create a platform technology for performing complex computing with a consortium of bacteria with a far-fetched dream towards bacterial machine intelligence. We believe the ANN type architecture with bacteria might help to achieve such a goal.

The gene regulation in bacteria is generally non-linear. The transcription factors are bound to the various operating sites of a promoter in dimer or multimer forms to regulate the transcription. A small molecule inducer or inhibitor binds to those transcription factors to activate or inhibit the transcription process. This results in a non-linear relationship between the inducer concentration and the protein produced from transcription and translation.^48,49^ However, proper thresholding is important to create a specific activation function with appropriate parameters. For thresholding and optimizing log sigmoid behavior in bactoneurons, we changed the numbers and relative positions of various promoter operator sites in our synthetic promoters, relative molecular amounts of transcription factors and promoter copy numbers through different plasmid copy numbers or altering the strength of ribosome binding by changing the nucleic acid sequence of the ribosome binding sites (RBS) in the promoter. This allowed change in both weight and bias parameters in the individual log sigmoid activation functions of the bactoneuron.

In order to use bactoneuron networks as a streamlined microbial computing platform, the bactoneuron must work robustly, stably and reproducibly within a given network. All the basic bactoneurons we created activated or interacted with several chemicals (with a non-zero weight value). However, within a network it experienced more chemicals (with zero weight value). We observed that the behavior of any specific bactoneuron did not change in various chemical environments (Fig. S5†). Second, the bactoneurons were modular and work in proper coordination with other bactoneurons within a network. Each bactoneuron carried a cellular device, which was physically separated by cell walls. As a result, their function did not interfere with others and there were no cross-talks between two cellular devices from different bactoneurons. Those were evident from the fact that a unit bactoneuron was part of many networks and they worked in proper coordination with other bactoneurons in various networks. For example, the unit bactoneuron BNeu 2 worked appropriately as a part of the demultiplexer, multiplexer, decoder, encoder, and Feynman gate (Fig. S1†). Similarly, BNeu 1 was part of the demultiplexer, decoder, and majority function (Fig. S1†). Those unit bactoneurons performed the coordinated tasks properly at least for 16 hours. However, the experiments were not done beyond 16 hours. In that context the stability of our circuits is maintained for at least 16 hours under induced conditions for all the bactoneurons (excluding the overnight plate and overnight uninduced liquid media culture). This timing was in the range of other genetic circuits^27,28,30–32,36^ developed in plasmids and propagated in bacteria. Furthermore, this stability is reproducible. We have performed several independent experiments (Table S8†) for optimized single unit bactoneurons individually as well as in a mixed population. Each of the bactoneuron was tested independently for fold change characterization, dose response behavior, validation experiments for simulation and within various mixed cultures for the full function characterization. The details of culture conditions, seeding, propagation, and number of experiments for each bactoneuron are tabulated in Table S8.† However, as the genome encoded synthetic genetic circuits are more stable than the plasmid encoded circuits,^50^ for long term functional stability of the bacteria-based ANN platform, the cellular device should be encoded in the bacterial genome.

One of the limitations of our study was that it did not explore the variation in the results due to probable variation in the growth rate of various sub-populations during cell multiplication^51^ and variation generated from the undefined LB media from experiment to experiment. It was interesting to note that with those plausible sources of variation the bactoneurons work reproducibly and stably within the experimental limit. However, studying such variation in the case of bactoneuron ANN would be an important future study. Furthermore, the dynamics of the bactoneuron would be an important property,^52^ which we did not explore in this study and it could be an important addition for near future study.

One of the advantages of creating biocomputing functions with bactoneuron ANNs, as we discussed earlier, was that it did not require layering of logic gates. Thus, we created a multiplexer and an encoder with a single layer bactoneuron ANN, though both of them consisted of at least three layers of logic gates in their simplest electronic design.

Increasing another hidden layer of artificial neurons may help to approximate a more complex function appropriately.^1^ Our near future goal is to increase a layer in our bactoneuron architectures by replacing our fluorescent reporter proteins with quorum-sensing genes, which produce diffusive quorum-sensing molecules and secrete to activate another bactoneuron at a distance.^30^

## Conclusions

In summary, we showed that the basic concept of ANN could be adapted in living bacteria with the help of cellular devices. The ANN type architecture with molecular engineered bacteria works as a flexible and general framework in its design and architecture for performing complex bio-computation, both conventional and reversible. Unlike the conventional *in vivo* synthetic genetic computing,^31,53^ which followed the hierarchical logic circuit approach,^16,54^ we showed that the ANN type framework can adapt a new design path to create complex computing functions like encoder, majority function, Mux, Demux, Feynman gate and Fredkin gate, where the encoder, majority function and reversible gates were demonstrated for the first time in living cells. Reversible computing is a new class of computing for biological cells and our work might pave the way in that direction. In ANNs, any function can be designed and simulated just by adjusting the weights and bias values of a single mathematical equation and we established a direct relationship between signs of the weights and nature of the interaction in the cellular devices. Thus, our approach established a new streamlined design and construction platform complementary to the conventional bio-circuit design^55^ and may have implications in complex reversible and irreversible biocomputing, bacteria-based ANN hardware, and synthetic biology.

## Materials and methods

### Promoters and genes, plasmids, RBSs & primers

The genetic devices were made according to the designs. The bioparts (promoter, ribosome binding sites (RBS), gene and transcription terminators) were arranged within appropriate plasmids using standard molecular biology protocols. PCR amplification was performed using KOD Hot Start DNA polymerase (Merck Millipore). All enzymes, ligase, and ladders were from New England BioLabs. Plasmid isolation, gel extraction, and PCR purification kits were from QIAGEN. The translation initiation rate for EGFP and CI under the control of the promoter P_AAH_ and P_LtetO-1_/P_LlacO-1_, respectively, was calculated and weak RBSs (RBSs RH and RC1-3) were designed using RBS Calculator v2.0,^56^ considering RBS R (BBa_B0034)^57^ along with linker GGTACC (*Kpn*I site) as the degenerate RBS sequence and *E. coli*-MG1655 as the organism. All promoter sequences, RBS sequences, primers, and plasmids are shown in ESI Tables S3 and S9.† All primers, oligos and gene products were obtained from IDT and Invitrogen. All cloned genes, promoters, and RBSs in plasmid constructs were sequence verified by Eurofins Genomics India Pvt. Ltd, Bangalore, India.

### Bacterial cell culture for characterization

Chemically competent *Escherichia coli* DH5α strain was used for cloning and DH5αZ1 strain was used for the experimental characterization. Working concentrations of the antibiotics in LB-Agar, Miller (Difco, Beckton Dickinson) plates as well as in LB broth, Miller (Difco, Beckton Dickinson) were: 100 μg ml^−1^ for ampicillin (Himedia), 34 μg ml^−1^ for chloramphenicol (Himedia) and 50 μg ml^−1^ for kanamycin (Sigma Aldrich). DH5αZ1 cells were transformed with appropriate sequence verified plasmid constructs. Well-isolated single colonies were picked from LB agar-plates, inoculated to fresh LB-liquid media, and grown overnight in the presence of antibiotics. Next, the overnight culture was re-diluted 100 times in fresh LB media with antibiotics and with or without inducers, as per the design of the gene circuit, and grown at 37 °C and ∼250 rpm. Engineered cells for weight and bias adjustment steps were grown for 6/12/16 hours (expression characterization and dose response experiments) and cells with final constructs were grown for 10 hours with inducers, resuspended and grown for another 6 hours. Such 10 + 6 hours growth was performed for expression characterization, dose response experiments, validation experiments and for full ANN microscopy experiments (Table S8†).

### Dose–response experiments and validation experiments

All dose–response experiments were performed by varying one inducer across 9 or more concentration points, while the other inducer was kept constant (“0” state or “1” as the case may be). Here, for the linear combinations of input signals, we converted the concentration range of each input chemical from ‘0’ to ‘1’, where 0 signifies zero concentration and 1 signifies the saturating concentration of the chemical. Any concentration higher than the saturation concentration was treated as 1. For the validation experiments, the corresponding two inducers of the relevant constructs were simultaneously varied across 10+ concentration points. For validation experiments, we used different concentration points compared to the concentration used in dose response. For intermediate experiments, during weight and bias adjustments of the constructs, a single colony was used. For the optimized constructs, all dose response and fold characterization experiment data from minimum 3 independent colonies were collected.

### Measurement of fluorescence and optical density, normalization, and scaling

For fluorescence and optical density (OD) measurements, a Synergy HTX Multi-Mode reader (Biotek Instruments, USA) was used. For this purpose, cells were diluted in PBS (pH 7.4) to reach around OD600 as 0.8, loaded onto a 96-well multi-well plate (black, Greiner Bio-One), and both EGFP fluorescence with appropriate gain and OD600 were measured. For EGFP fluorescence measurements, we used a 485/20 nm excitation filter and 516/20 nm emission bandpass filter. At least 3 biological replicates were considered for each condition to collect the fluorescence and OD data. The raw fluorescence values were divided by the respective OD600 values and thus normalized to the number of cells. Auto-fluorescence was measured as average normalized fluorescence of the untransformed DH5αZ1 set (no plasmid set) and subtracted from the normalized fluorescence value of the experimental set. The above normalization can be mathematically represented as follows:
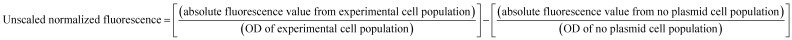


The values thus obtained were then scaled down between 0 and 1; considering the normalized fluorescence value at the induction point of maximum expected fluorescence to be 1,
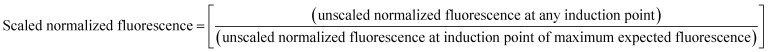


### Data analysis, fitting, mathematical modeling and simulation

The fittings of the dose response curves were done using appropriate equations (ESI Table S1†). However, all the equations could be brought down to a general form:2
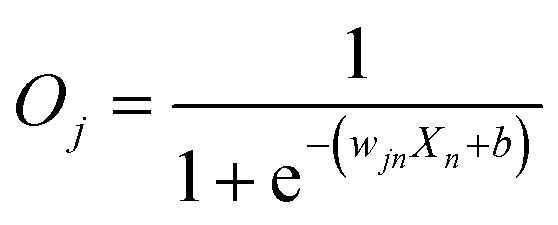
where *O*_*j*_ is the output signal from the artificial neuron *j*, *X*_*n*_ corresponds to the magnitude of the varying input (*n*) to the artificial neuron *j*, *w*_*n*_ corresponds to the weight of the *n*^th^ input to the artificial neuron *j*, and *b* is given by:

3 *b* = *X*_*m*_*w*_*jm*_ + *b*_*j*_

where *X*_*m*_ corresponds to the logical state of the constant input (*m*) to the artificial neuron *j* (0 or 1), *w*_*m*_ corresponds to the weight of the *m*^th^ input to the artificial neuron *j*, and *b*_*j*_ corresponds to the bias of the artificial neuron *j*.

The scaled output fluorescence values obtained from the dose–response experiments were plotted against the varying inducer concentration and fitted against eqn (2). All data analysis and fitting were performed in OriginPro 2018 (OriginLab Corporation, USA) and using a built-in Levenberg Marquardt algorithm, a damped least squares (DLS) method. The parameter “*w*_*jn*_” of the fitting function (eqn (2)) gives the “weight” of the varying input in the summation function of the corresponding neuron j. The *b* value obtained includes the bias plus the product of the input logic state of the second input and its weight as explained above. Upon similarly fitting the dose–response of the neuron to the second input, the weight “*w*_*j*_” and “*b*” for input 2 is obtained. Solving eqn (2) and (3) for both the inputs, “*w*_*j*input1_”, “*w*_*j*input2_” and “*b*_*j*_” of the complete summation function of the corresponding neuron *j* were obtained. All parameter values for all constructs are shown in ESI Table S4.† The simulations were performed by generating matrices of calculated, normalized output fluorescence values against simultaneously varying concentrations of the corresponding two inputs across 65 × 65 or more points, following the parameterized activation function. For single-input systems, simulation for a given activation function was carried out across 19 varying input concentration points.

### Microscopy

DH5αZ1 cells were transformed with the appropriate sequence-verified plasmid construct(s). Following a 10 hour induction followed by re-suspension and 6 hour induction step, cells were washed thrice in PBS. Cell pellets were finally resuspended in fresh PBS (pH 7.2–7.4) and this re-suspension was used to prepare fresh slides. A Laser Scanning Microscope Zeiss LSM 710/ConfoCor 3 operating on ZEN 2008 software was used for imaging the de-multiplexer, multiplexer, majority function, decoder, encoder and Feynman gate. The cell suspension slides were subjected to excitation by appropriate laser channels (458 nm Ar Laser for mTFP1, 488 nm Ar Laser for EGFP, 514 nm Ar Laser for mVenus, 543 nm He–Ne Laser for mKO2 and 633 nm He–Ne Laser for E2-Crimson) and fluorescence emissions were captured through suitable emission filters (BP484-504 nm for mTFP1, BP500-520 nm for EGFP, BP521-541 nm for mVenus, BP 561–591 nm for mKO2, and BP641-670 nm (2-to-4 decoder)/BP630-650 nm (1-to-2 de-multiplexer) for E2-Crimson) with a 63× oil immersion objective and were detected through a T-PMT. The pin hole was completely open. For reversible Fredkin gate imaging, a Nikon AIR Si confocal microscope along with a resonant scanner and coherent CUBE diode laser system was used. The mixed cell population, washed and resuspended in PBS (pH 7.2–7.4), was added on the top of a 1% molten agarose pad, which was placed upon a cleaned glass slide. The sample field was then covered with a clear cover slip, placed under 60× water immersion and subjected to excitation by laser channels (488 nm laser for EGFP, 561 nm for td-Tomato and 640 nm for E2-Crimson). Three different emission filters (BP 525/50 nm for EGFP, BP 585/65 for td-Tomato and BP 700/75 nm for E2-Crimson) were used for measuring the fluorescence. Differential interference contrast (DIC) images were captured for all samples as well. Microscopic images were processed through ImageJ software for better visualization.

## Data availability

Data are available within the article or in the ESI file.†

## Author contributions

SB conceived and designed the study. KS, DB, and RS performed all the experiments. KS, DB, and SB designed the experiments, analyzed and interpreted the data, and wrote the paper.

## Conflicts of interest

We have no competing interest.

## Supplementary Material

SC-012-D1SC01505B-s001
