# Supplementary material for: A single layer artificial neural network type architecture with molecular engineered bacteria for reversible and irreversible computing
Source: Chem Sci. 2021 Nov 9;12(48):15821–32. doi: 10.1039/d1sc01505b (PMC8672730; doi:10.1039/d1sc01505b)
Supplement: SC-012-D1SC01505B-s001 [file SC-012-D1SC01505B-s001.pdf]

Electronic Supplementary Material (ESI) for Chemical Science.
This journal is © The Royal Society of Chemistry 2021

Supplementary Information

A single layer artificial neural network type architecture with molecular engineered bacteria for reversible and irreversible computing

Kathakali Sarkar[1,2], Deepro Bonnerjee[1,2], Rajkamal Srivastava[1,2], Sangram Bagh[1,*]

[1]Biophysics and Structural Genomics Division, Saha Institute of Nuclear Physics, Homi Bhabha National Institute (HBNI), Block A/F, Sector-I, Bidhannagar, Kolkata 700064 INDIA
[2] Equal contributions
* Corresponding author

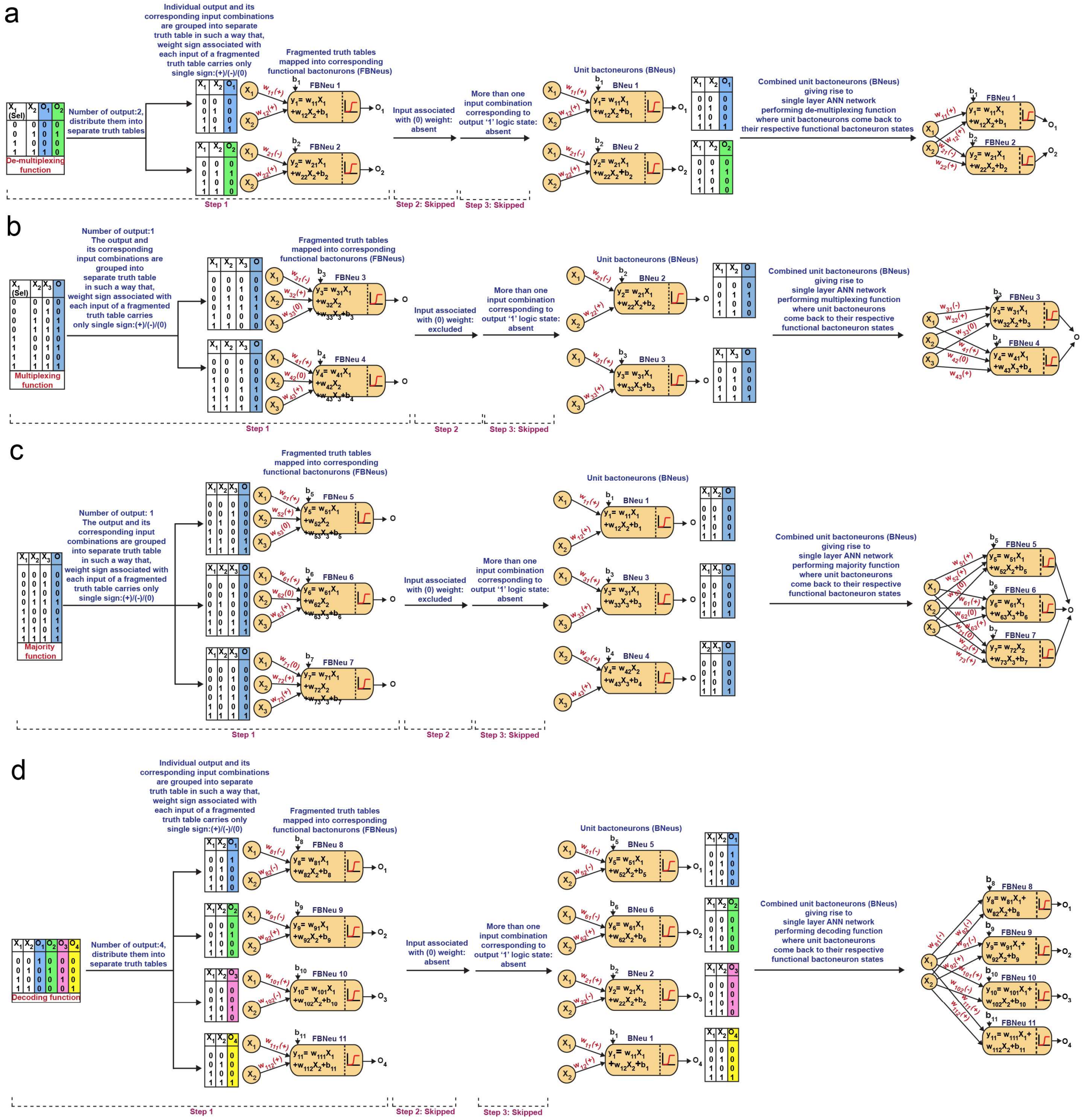
a
Individual output and its corresponding input combinations are grouped into separate truth table in such a way that, weight sign associated with each input of a fragmented truth table carries only single sign:(+)/(-)/(0)
Fragmented truth tables mapped into corresponding functional bactonurons (FBNeus)
Number of output:2, distribute them into separate truth tables
De-multiplexing function
FBNeu 1
FBNeu 2
Input associated with (0) weight: absent
More than one input combination corresponding to output '1' logic state: absent
Unit bactoneurons (BNeus)
BNeu 1
BNeu 2
Combined unit bactoneurons (BNeus) giving rise to single layer ANN network performing de-multiplexing function where unit bactoneurons come back to their respective functional bactoneuron states
Step 1
Step 2: Skipped
Step 3: Skipped
b
Number of output:1 The output and its corresponding input combinations are grouped into separate truth table in such a way that, weight sign associated with each input of a fragmented truth table carries only single sign:(+)/(-)/(0)
Multiplexing function
Fragmented truth tables mapped into corresponding functional bactonurons (FBNeus)
FBNeu 3
FBNeu 4
Input associated with (0) weight: excluded
More than one input combination corresponding to output '1' logic state: absent
Unit bactoneurons (BNeus)
BNeu 2
BNeu 3
Combined unit bactoneurons (BNeus) giving rise to single layer ANN network performing multiplexing function where unit bactoneurons come back to their respective functional bactoneuron states
Step 1
Step 2
Step 3: Skipped
c
Fragmented truth tables mapped into corresponding functional bactonurons (FBNeus)
Number of output: 1 The output and its corresponding input combinations are grouped into separate truth table in such a way that, weight sign associated with each input of a fragmented truth table carries only single sign:(+)/(-)/(0)
Majority function
FBNeu 5
FBNeu 6
FBNeu 7
Input associated with (0) weight: excluded
More than one input combination corresponding to output '1' logic state: absent
Unit bactoneurons (BNeus)
BNeu 1
BNeu 3
BNeu 4
Combined unit bactoneurons (BNeus) giving rise to single layer ANN network performing majority function where unit bactoneurons come back to their respective functional bactoneuron states
Step 1
Step 2
Step 3: Skipped
d
Individual output and its corresponding input combinations are grouped into separate truth table in such a way that, weight sign associated with each input of a fragmented truth table carries only single sign:(+)/(-)/(0)
Fragmented truth tables mapped into corresponding functional bactonurons (FBNeus)
Number of output:4, distribute them into separate truth tables
Decoding function
FBNeu 8
FBNeu 9
FBNeu 10
FBNeu 11
Input associated with (0) weight: absent
More than one input combination corresponding to output '1' logic state: absent
Unit bactoneurons (BNeus)
BNeu 5
BNeu 6
BNeu 2
BNeu 1
Combined unit bactoneurons (BNeus) giving rise to single layer ANN network performing decoding function where unit bactoneurons come back to their respective functional bactoneuron states
Step 1
Step 2: Skipped
Step 3: Skipped

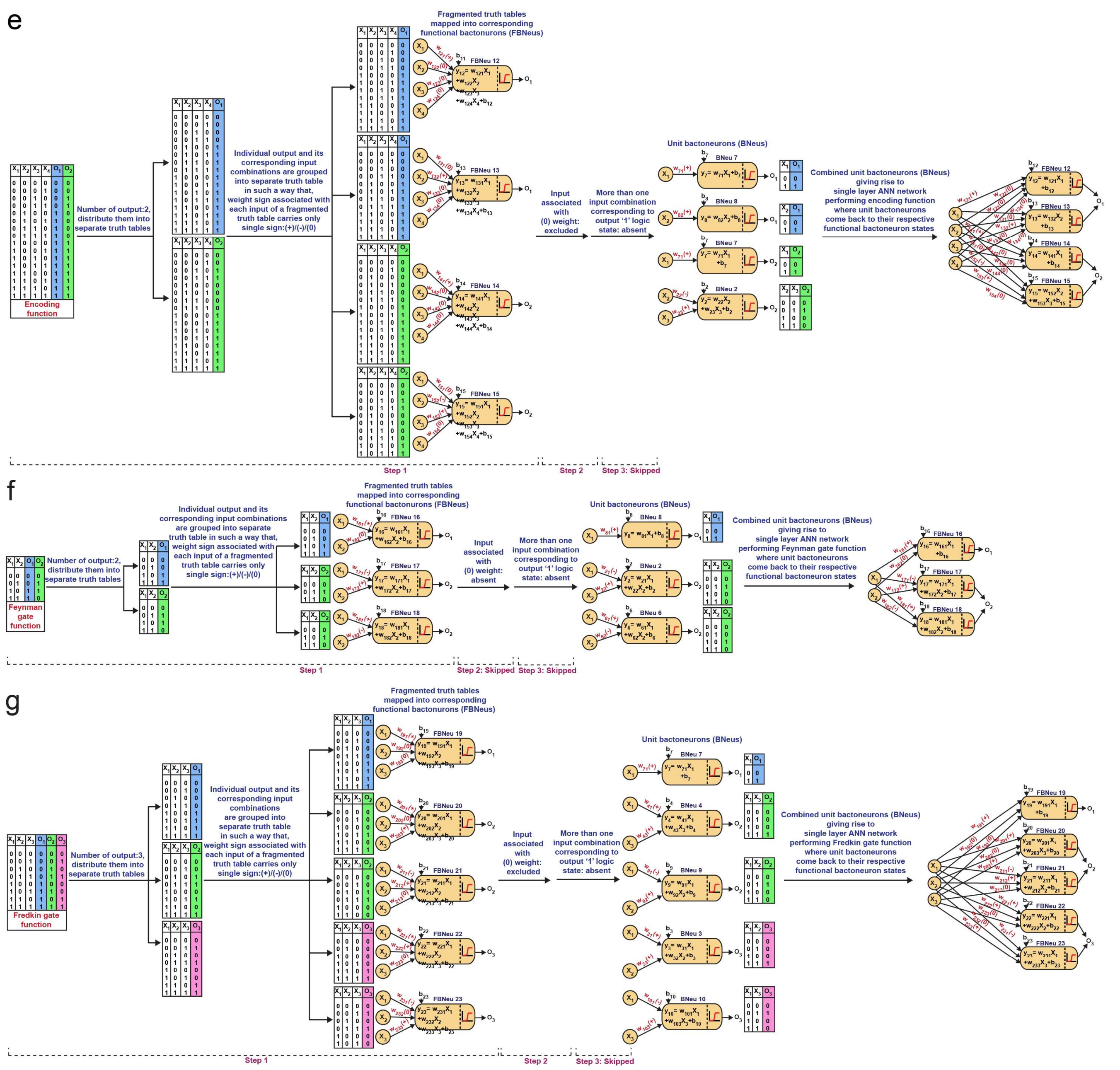

Figure S1: Derivation of functional and unit bactoneurons for a) de-multiplexing function, b) multiplexing function, c) majority function, d) decoding function, e) encoding function, f) Feynman gate function and g) Fredkin gate function. In each case, combination of unit bactoneurons gives rise to single layer ANN architecture where, individual unit bactoneurons come back to their corresponding functional bactoneuron states while they get associated with '0' weighted inputs (If any). In the network level, parts of the summation function of each functional bactoneuron, contributed by '0' weighted input, are not shown.

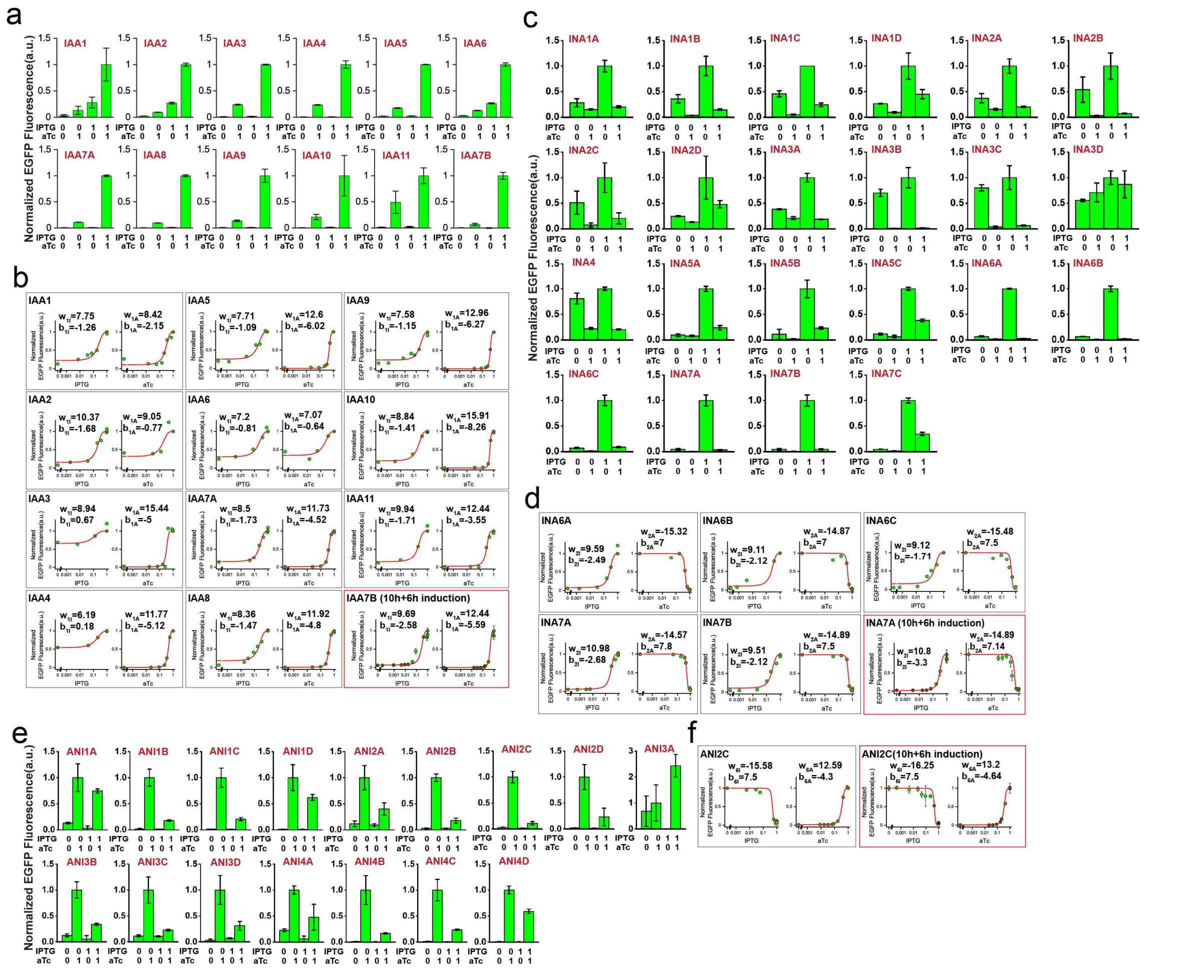

Figure S2: Details of characterization and dose responses of BNeus 1, 2 and 6. Expression characterization of **a)** BNeu 1, **c)** BNeu 2 and **e)** BNeu 6 and dose responses of **b)** BNeu 1, **d)** BNeu 2 and **f)** BNeu 6.

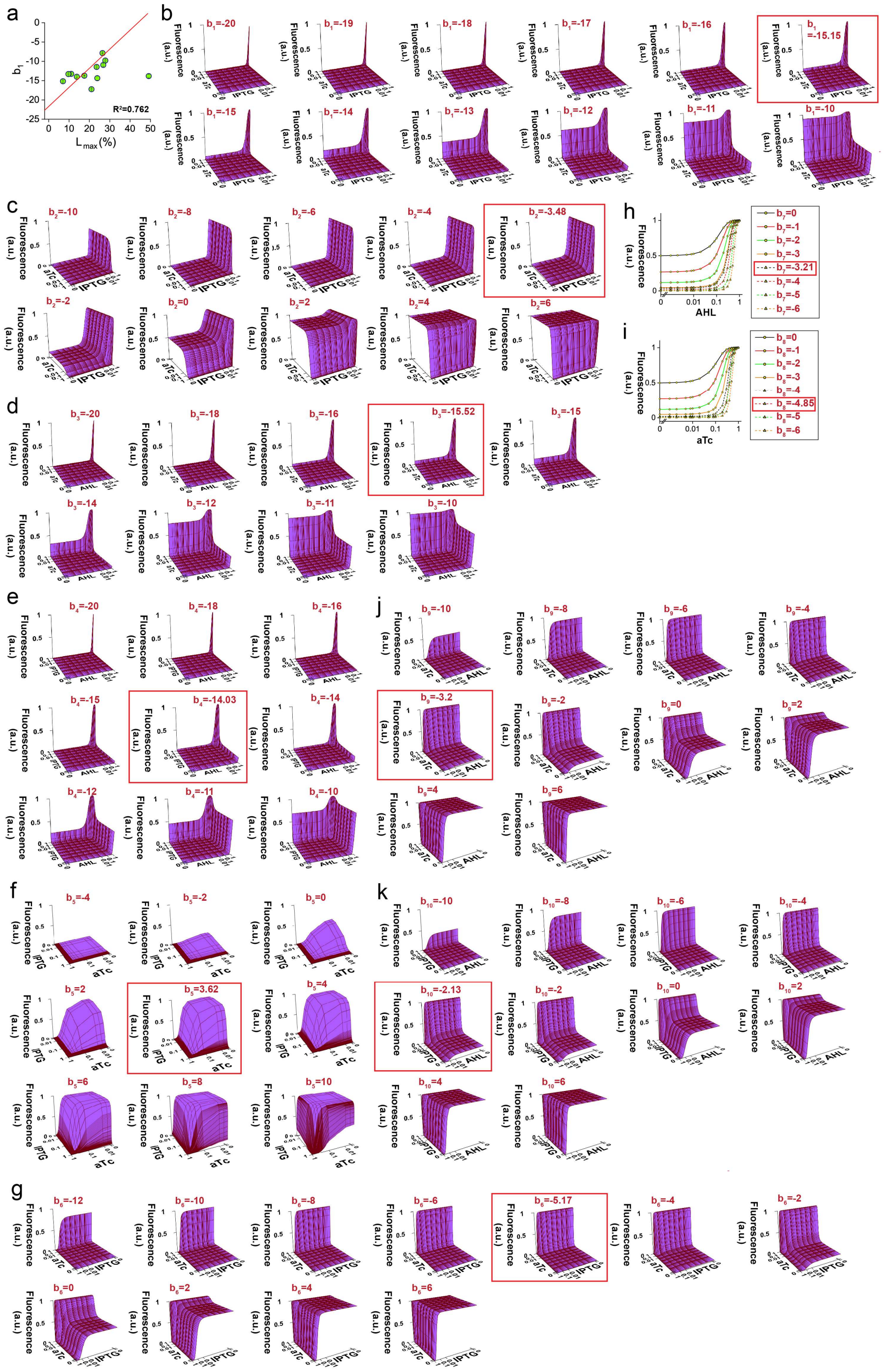

Figure S3: Correlation between bias and leakage of a Unit bactoneuron. a) Correlation between bias (b_1) and the percentage highest leakage (L_{max}(%)) for all BNeu 1 cellular devices obtained from weight and bias adjustment steps. Simulated output behaviors of **b)** BNeu 1, **c)** BNeu 2 **d)** BNeu 3 **e)** BNeu 4, **f)** BNeu 5, **g)** BNeu 6, **h)** BNeu 7, **i)** BNeu 8, **j)** BNeu 9 and **k)** BNeu 10. Simulation corresponding to the bias value obtained experimentally for each bactoneuron is shown in red box.

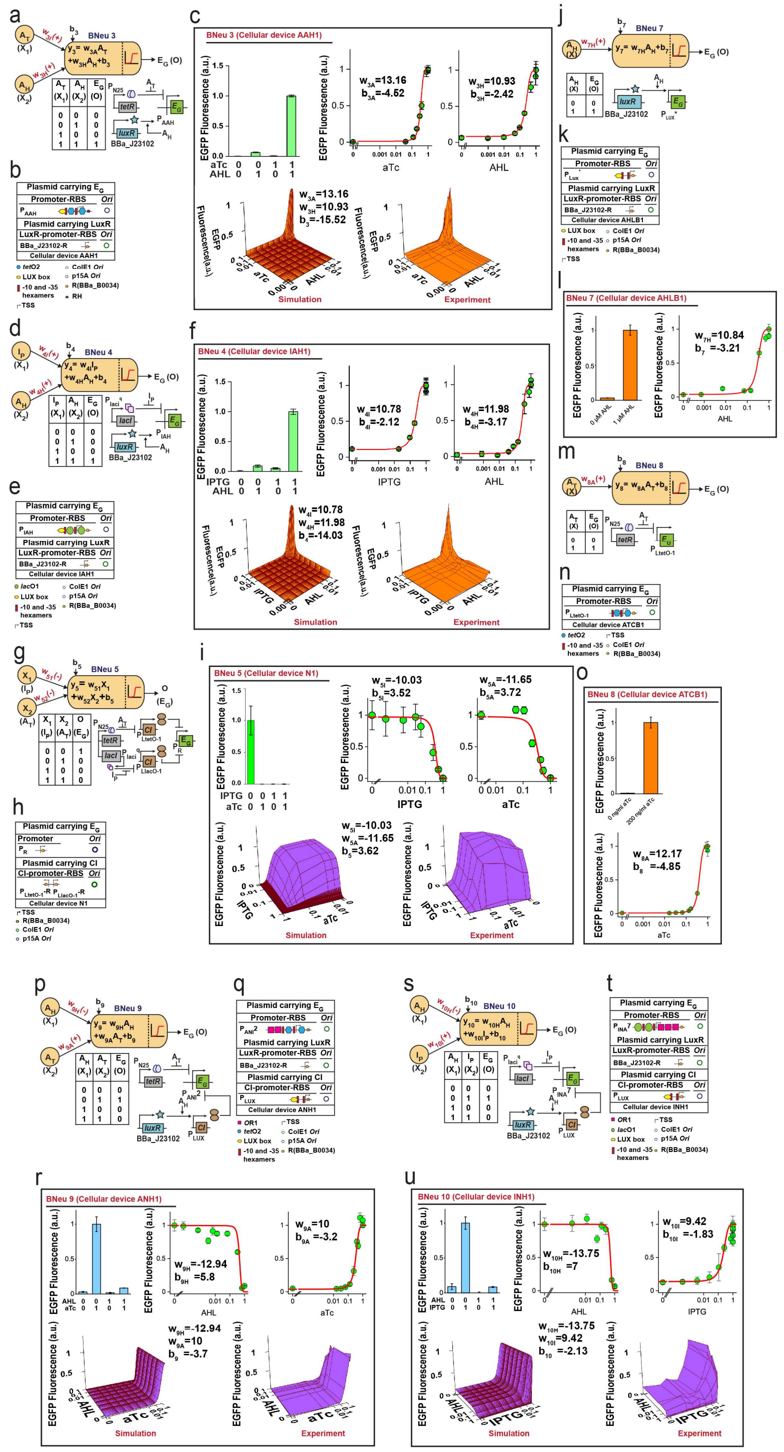

Figure S4: Characterization of unit bactoneurons BNeu 3, BNeu 4, BNeu 5, BNeu 7, BNeu 8, BNeu 9 and BNeu 10. Neural architectures, truth tables and biological circuit designs of unit bactoneurons **a)** BNeu 3, **d)** BNeu 4, **g)** BNeu 5, **j)** BNeu 7, **m)** BNeu 8, **p)** BNeu 9 and **s)** BNeu 10 are shown. Details of plasmids carrying bioparts of the biological circuit designs of **b)** BNeu 3, **e)** BNeu 4, **h)** BNeu 5, **k)** BNeu 7, **n)** BNeu 8, **q)** BNeu 9 and **t)** BNeu 10 are illustrated. Expression characterization, dose responses, 3D simulations and experimental 3D behavior of **c)**BNeu3, **f)** BNeu 4, **i)** BNeu 5, **l)** BNeu 7, **o)** BNeu 8, **r)** BNeu 9 and **u)** BNeu 10 in terms of EGFP expression are also shown.

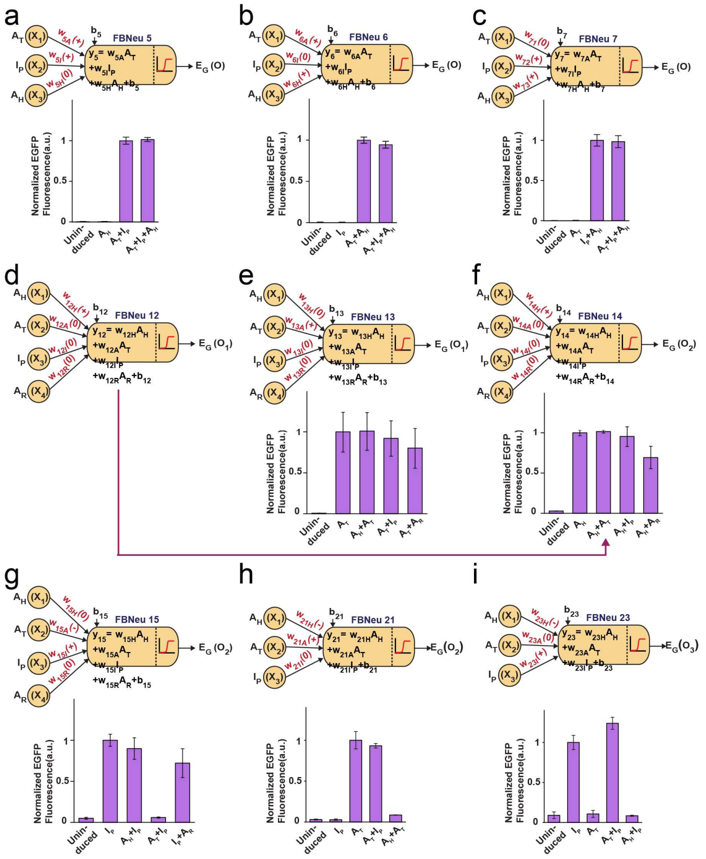

Figure S5: Characterizations of functional bactoneurons associated with weight=0 towards specific inducers. Each functional bactoneuron population was subjected to 10h+6h induction with a set of inducers which was chosen based on the neural architecture of individual functional bactoneurons, and then characterized in terms of EGFP expression. If the presence or absence of a specific inducer didn't change the output of the functional bactoneuron, then only we considered that, the inducer was associated with zero weight.Neural architecturesand validation for '0' weighted input(s) of functional bactoneurons**a)**FBNeu 5, **b)** FBNeu 6, **c)** FBNeu 7, **d)** FBNeu 12, **e)** FBNeu 13, **f)** FBNeu 14, **g)** FBNeu 15, **h)** FBNeu 21 and **i)** FBNeu 23 are shown. FBNeu 20 and FBNeu 22 from Fredkin gate function are equivalent to FBNeu 7 and FBNeu 6 respectively except their different outputs. Therefore, individual weight '0' input validation for FBNeus 6 and 7 justifies the same for FBNeus 22 and 20 as well. FBNeu 12 and FBNeu 14 are similar except their outputs. Here, individual functional bactoneurons are characterized in terms of EGFP output. Therefore, both FBNeu 12 and FBNeu 14 produce EGFP output and hence, they become identical. Thus, they share common '0' weighted input validation data (Shown with magenta arrow). FBNeu 16 from Feynman gate function is a sub-set of FBNeu 13 as it operates on lesser number of inputs whereas, their corresponding unit bactoneuron is common. Therefore, weight '0' input of FBNeu 16 can be validated from the characterization result of FBNeu 13. Similarly, weight '0' inputs of Fredkin gate functional bactoneuron FBNeu 19 can be validated by characterization result of FBNeu 12/14.

ab

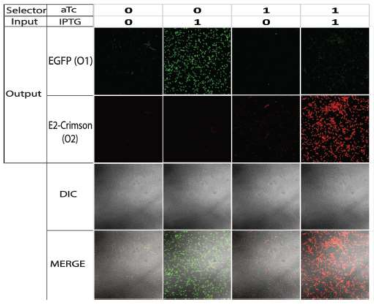

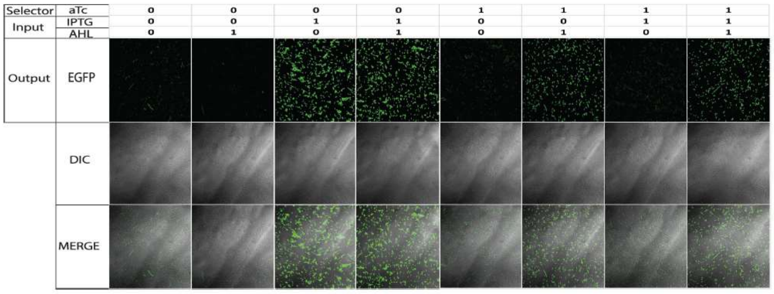

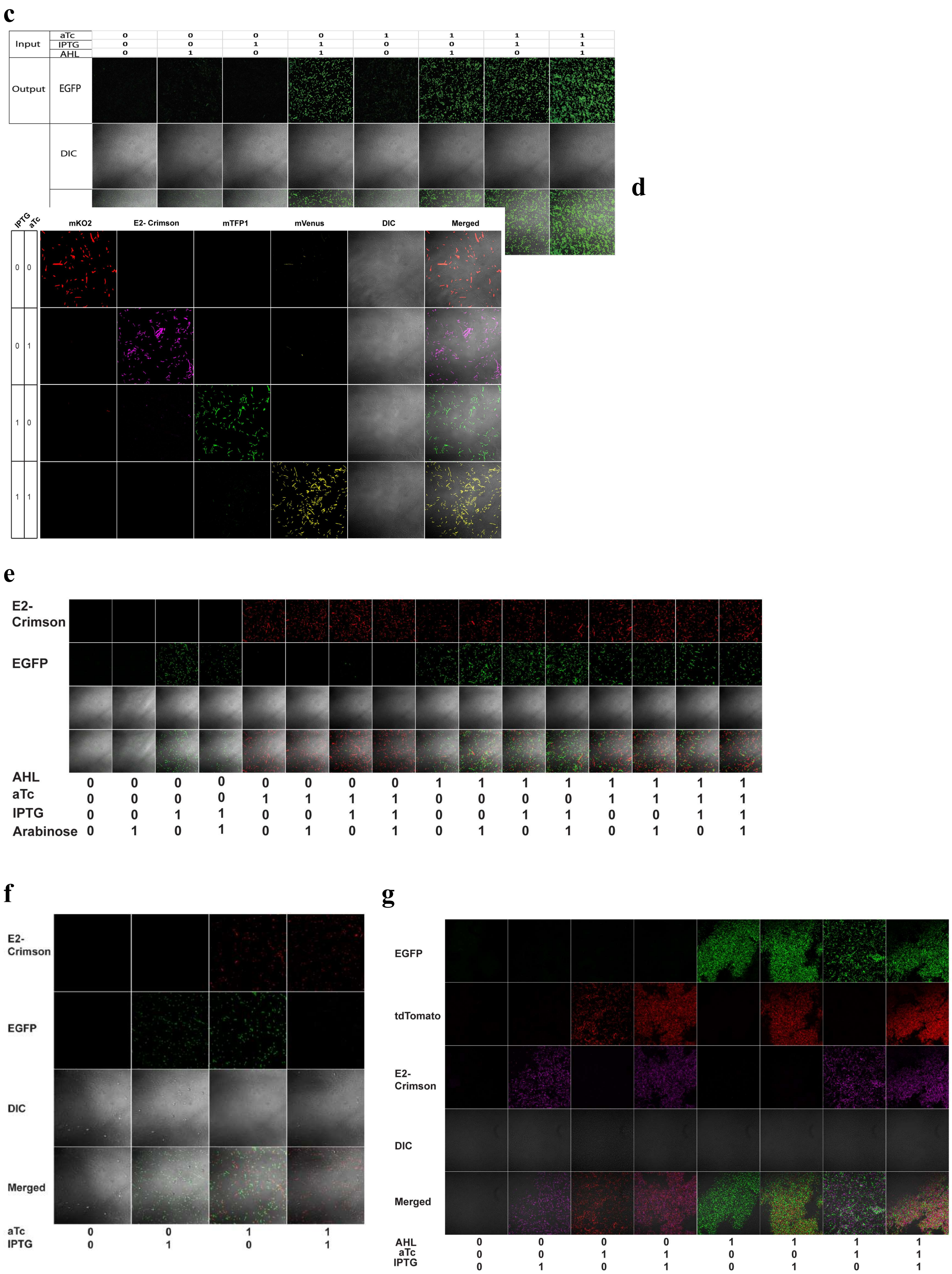

Figure S6: Microscopy images with corresponding differential interference contrast (DIC) and merged channels. Populations of different combinations of bactoneurons, depending on the complex functions they constitute, were co-cultured with appropriate inductions where, they together formed a bactoneural layer. They were viewed under relevant laser channels and emission filters. DIC images show a heterogenous population of cells in the field with each sub-population responding uniquely to the induction conditions. A bactoneuron's activation is reported by fluorescence from its respective output protein

whereas inactive bactoneurons show no fluorescence. Microscopic images for the bactoneuron-based single layer ANN type architectures for **a)** de-multiplexing function, **b)** multiplexing function, **c)** majority function, **d)** decoding function, **e)** encoding function, **f**) reversible Feynman gate and **g)** reversible Fredkin gate are shown.

Table S1: Details of functional bactoneurons and corresponding unit bactoneurons associated with the computing functions developed in this study. Output fluorescent proteins and activation function equations corresponding to unit bactoneurons are also described**.**

Serial number	Function	Functional bactoneurons (FBNeus)	Unit bactoneurons (BNeus)	Output of unit bactoneuron	Activation function Equation
1	De-multiplexing	FBNeu 1: X_1 (A_T), X_2 (I_P); $w_{1A}(+)$, $w_{1I}(+)$; $y_1 = w_{1A}A_T + w_{1I}I_P + b_1$ → O_1 (E_2)	BNeu 1: A_T, I_P; $w_{1A}(+)$, $w_{1I}(+)$; $y_1 = w_{1A}A_T + w_{1I}I_P + b_1$ → E_G /E_2 /M_V	E2-Crimson (E_2)	$O_1 = \frac{1}{1+e^{-(w_{1I}\cdot I_P + w_{1A}\cdot A_T + b_1)}}$
		FBNeu 2: X_1 (A_T), X_2 (I_P); $w_{2A}(-)$, $w_{2I}(+)$; $y_2 = w_{2A}A_T + w_{2I}I_P + b_2$ → O_2 (E_G)	BNeu 2: A_T, I_P; $w_{2A}(-)$, $w_{2I}(+)$; $y_2 = w_{2A}A_T + w_{2I}I_P + b_2$ → E_G /M_T	EGFP (E_G)	$O_2 = \frac{1}{1+e^{-(w_{2I}\cdot I_P + w_{2A}\cdot A_T + b_2)}}$
2	Multiplexing	FBNeu 3: X_1 (A_T), X_2 (I_P), X_3 (A_H); $w_{3A}(-)$, $w_{3I}(+)$, $w_{3H}(0)$; $y_3 = w_{3A}A_T + w_{3I}I_P + w_{3H}A_H + b_3$ → O (E_G)	BNeu 2: A_T, I_P; $w_{2A}(-)$, $w_{2I}(+)$; $y_2 = w_{2A}A_T + w_{2I}I_P + b_2$ → E_G /M_T	EGFP (E_G)	$O_2 = \frac{1}{1+e^{-(w_{2I}\cdot I_P + w_{2A}\cdot A_T + b_2)}}$
		FBNeu 4: X_1 (A_T), X_2 (I_P), X_3 (A_H); $w_{4A}(+)$, $w_{4I}(0)$, $w_{4H}(+)$; $y_4 = w_{4A}A_T + w_{4I}I_P + w_{4H}A_H + b_4$ → O (E_G)	BNeu 3: A_T, A_H; $w_{3A}(+)$, $w_{3H}(+)$; $y_3 = w_{3A}A_T + w_{3H}A_H + b_3$ → E_G /E_2	EGFP (E_G)	$O_3 = \frac{1}{1+e^{-(w_{3A}\cdot A_T + w_{3H}\cdot A_H + b}}$
3	Majority function	FBNeu 5: X_1 (A_T), X_2 (I_P), X_3 (A_H); $w_{5A}(+)$, $w_{5I}(+)$, $w_{5H}(0)$; $y_5 = w_{5A}A_T + w_{5I}I_P + w_{5H}A_H + b_5$ → O (E_G)	BNeu 1: A_T, I_P; $w_{1A}(+)$, $w_{1I}(+)$; $y_1 = w_{1A}A_T + w_{1I}I_P + b_1$ → E_G /E_2 /M_V	EGFP (E_G)	$O_1 = \frac{1}{1+e^{-(w_{1I}\cdot I_P + w_{1A}\cdot A_T + b_1)}}$
		FBNeu 6: X_1 (A_T), X_2 (I_P), X_3 (A_H); $w_{6A}(+)$, $w_{6I}(0)$, $w_{6H}(+)$; $y_6 = w_{6A}A_T + w_{6I}I_P + w_{6H}A_H + b_6$ → O (E_G)	BNeu 3: A_T, A_H; $w_{3A}(+)$, $w_{3H}(+)$; $y_3 = w_{3A}A_T + w_{3H}A_H + b_3$ → E_G /E_2	EGFP (E_G)	$O_3 = \frac{1}{1+e^{-(w_{3A}\cdot A_T + w_{3H}\cdot A_H + b}}$
		FBNeu 7: X_1 (A_T), X_2 (I_P), X_3 (A_H); $w_{7A}(0)$, $w_{7I}(+)$, $w_{7H}(+)$; $y_7 = w_{7A}A_T + w_{7I}I_P + w_{7H}A_H + b_7$ → O (E_G)	BNeu 4: I_P, A_H; $w_{4I}(+)$, $w_{4H}(+)$; $y_4 = w_{4I}I_P + w_{4H}A_H + b_4$ → E_G /T_M	EGFP (E_G)	$O_4 = \frac{1}{1+e^{-(w_{4I}\cdot I_P + w_{4H}\cdot A_H + b_4)}}$
4	Decoding	FBNeu 8: X_1 (I_P), X_2 (A_T); $w_{8I}(-)$, $w_{8A}(-)$; $y_8 = w_{8I}I_P + w_{8A}A_T + b_8$ → O_1 (M_K)	BNeu 5: I_P, A_T; $w_{5I}(-)$, $w_{5A}(-)$; $y_5 = w_{5I}I_P + w_{5A}A_T + b_5$ → E_G /M_K	mKO2 (M_K)	$O_5 = \frac{1}{1+e^{-(w_{5I}\cdot I_P + w_{5A}\cdot A_T + b_5)}}$
5	Encoding	FBNeu 9: X_1 (I_P), X_2 (A_T); $w_{9I}(-)$, $w_{9A}(+)$; $y_9 = w_{9I}I_P + w_{9A}A_T + b_9$ → O_2 (E_2)	BNeu 6: I_P, A_T; $w_{6I}(-)$, $w_{6A}(+)$; $y_6 = w_{6I}I_P + w_{6A}A_T + b_6$ → E_G /E_2	E2-Crimson (E_2)	$O_6 = \frac{1}{1+e^{-(w_{6I}\cdot I_P + w_{6A}\cdot A_T + b_6)}}$
		FBNeu 10: X_1 (I_P), X_2 (A_T); $w_{10I}(+)$, $w_{10A}(-)$; $y_{10} = w_{10I}I_P + w_{10A}A_T + b_{10}$ → O_3 (M_T)	BNeu 2: I_P, A_T; $w_{2I}(+)$, $w_{2A}(-)$; $y_2 = w_{2I}I_P + w_{2A}A_T + b_2$ → E_G /M_T	mTFP1 (M_T)	$O_2 = \frac{1}{1+e^{-(w_{2I}\cdot I_P + w_{2A}\cdot A_T + b_2)}}$
		FBNeu 11: X_1 (I_P), X_2 (A_T); $w_{11I}(+)$, $w_{11A}(+)$; $y_{11} = w_{11I}I_P + w_{11A}A_T + b_{11}$ → O_4 (M_V)	BNeu 1: I_P, A_T; $w_{1I}(+)$, $w_{1A}(+)$; $y_1 = w_{1I}I_P + w_{1A}A_T + b_1$ → E_G /E_2 /M_V	mVenus (M_V)	$O_1 = \frac{1}{1+e^{-(w_{1I}\cdot I_P + w_{1A}\cdot A_T + b_1)}}$
		FBNeu 12: X_1 (A_H), X_2 (A_T), X_3 (I_P), X_4 (A_R); $w_{12H}(+)$, $w_{12A}(0)$, $w_{12I}(0)$, $w_{12R}(0)$; $y_{12} = w_{12H}A_H + w_{12A}A_T + w_{12I}I_P + w_{12R}A_R + b_{12}$ → O_1 (E_2)	BNeu 7: A_H; $w_{7H}(+)$; $y_7 = w_{7H}A_H + b_7$ → E_G /E_2	E2-Crimson (E_2)	$O_7 = \frac{1}{1+e^{-(w_{7H}\cdot A_H + b_7)}}$
		FBNeu 13: X_1 (A_H), X_2 (A_T), X_3 (I_P), X_4 (A_R); $w_{13H}(0)$, $w_{13A}(+)$, $w_{13I}(0)$, $w_{13R}(0)$; $y_{13} = w_{13H}A_H + w_{13A}A_T + w_{13I}I_P + w_{13R}A_R + b_{13}$ → O_1 (E_2)	BNeu 8: A_T; $w_{8A}(+)$; $y_8 = w_{8A}A_T + b_8$ → E_G /E_2	E2-Crimson (E_2)	$O_8 = \frac{1}{1+e^{-(w_{8A}\cdot A_T + b_8)}}$
		FBNeu 14: X_1 (A_H), X_2 (A_T), X_3 (I_P), X_4 (A_R); $w_{14H}(+)$, $w_{14A}(0)$, $w_{14I}(0)$, $w_{14R}(0)$; $y_{14} = w_{14H}A_H + w_{14A}A_T + w_{14I}I_P + w_{14R}A_R + b_{14}$ → O_2 (E_G)	BNeu 7: A_H; $w_{7H}(+)$; $y_7 = w_{7H}A_H + b_7$ → E_G /E_2	EGFP (E_G)	$O_7 = \frac{1}{1+e^{-(w_{7H}\cdot A_H + b_7)}}$
		FBNeu 15: X_1 (A_H), X_2 (A_T), X_3 (I_P), X_4 (A_R); $w_{15H}(0)$, $w_{15A}(-)$, $w_{15I}(+)$, $w_{15R}(0)$; $y_{15} = w_{15H}A_H + w_{15A}A_T + w_{15I}I_P + w_{15R}A_R + b_{15}$ → O_2 (E_G)	BNeu 2: A_T, I_P; $w_{2A}(-)$, $w_{2I}(+)$; $y_2 = w_{2A}A_T + w_{2I}I_P + b_2$ → E_G /M_T	EGFP (E_G)	$O_2 = \frac{1}{1+e^{-(w_{2I}\cdot I_P + w_{2A}\cdot A_T + b_2)}}$
6	Feynman gate	FBNeu 16: X_1 (A_T), X_2 (I_P); $w_{16A}(+)$, $w_{16I}(0)$; $y_{16} = w_{16A}A_T + w_{16I}I_P + b_{16}$ → O_1 (E_2)	BNeu 8: A_T; $w_{8A}(+)$; $y_8 = w_{8A}A_T + b_8$ → E_G /E_2	E2-Crimson (E_2)	$O_8 = \frac{1}{1+e^{-(w_{8A}\cdot A_T + b_8)}}$
		FBNeu 17: X_1 (A_T), X_2 (I_P); $w_{17A}(-)$, $w_{17I}(+)$; $y_{17} = w_{17A}A_T + w_{17I}I_P + b_{17}$ → O_2 (E_G)	BNeu 2: A_T, I_P; $w_{2A}(-)$, $w_{2I}(+)$; $y_2 = w_{2A}A_T + w_{2I}I_P + b_2$ → E_G /M_T	EGFP (E_G)	$O_2 = \frac{1}{1+e^{-(w_{2I}\cdot I_P + w_{2A}\cdot A_T + b_2)}}$
		FBNeu 18: X_1 (A_T), X_2 (I_P); $w_{18A}(+)$, $w_{18I}(-)$; $y_{18} = w_{18A}A_T + w_{18I}I_P + b_{18}$ → O_2 (E_G)	BNeu 6: A_T, I_P; $w_{6A}(+)$, $w_{6I}(-)$; $y_6 = w_{6A}A_T + w_{6I}I_P + b_6$ → E_G /E_2	EGFP (E_G)	$O_6 = \frac{1}{1+e^{-(w_{6I}\cdot I_P + w_{6A}\cdot A_T + b_6)}}$
7	Fredkin gate	FBNeu 19: X_1 (A_H), X_2 (A_T), X_3 (I_P); $w_{19H}(+)$, $w_{19A}(0)$, $w_{19I}(0)$; $y_{19} = w_{19H}A_H + w_{19A}A_T + w_{19I}I_P + b_{19}$ → O_1 (E_G)	BNeu 7: A_H; $w_{7H}(+)$; $y_7 = w_{7H}A_H + b_7$ → E_G /E_2	EGFP (E_G)	$O_7 = \frac{1}{1+e^{-(w_{7H}\cdot A_H + b_7)}}$
		FBNeu 20: X_1 (A_H), X_2 (A_T), X_3 (I_P); $w_{20H}(+)$, $w_{20A}(0)$, $w_{20I}(+)$; $y_{20} = w_{20H}A_H + w_{20A}A_T + w_{20I}I_P + b_{20}$ → O_2 (T_M)	BNeu 4: A_H, I_P; $w_{4H}(+)$, $w_{4I}(+)$; $y_4 = w_{4H}A_H + w_{4I}I_P + b_4$ → E_G /T_M	tdTomato (T_M)	$O_4 = \frac{1}{1+e^{-(w_{4I}\cdot I_P + w_{4H}\cdot A_H + b_4)}}$
		FBNeu 21: X_1 (A_H), X_2 (A_T), X_3 (I_P); $w_{21H}(-)$, $w_{21A}(+)$, $w_{21I}(0)$; $y_{21} = w_{21H}A_H + w_{21A}A_T + w_{21I}I_P + b_{21}$ → O_2 (T_M)	BNeu 9: A_H, A_T; $w_{9H}(-)$, $w_{9A}(+)$; $y_9 = w_{9H}A_H + w_{9A}A_T + b_9$ → E_G /T_M	tdTomato (T_M)	$O_9 = \frac{1}{1+e^{-(w_{9A}\cdot A_T + w_{9H}\cdot A_H + b}}$
		FBNeu 22: X_1 (A_H), X_2 (A_T), X_3 (I_P); $w_{22H}(+)$, $w_{22A}(+)$, $w_{22I}(0)$; $y_{22} = w_{22H}A_H + w_{22A}A_T + w_{22I}I_P + b_{22}$ → O_3 (E_2)	BNeu 3: A_H, A_T; $w_{3H}(+)$, $w_{3A}(+)$; $y_3 = w_{3H}A_H + w_{3A}A_T + b_3$ → E_G /E_2	E2-Crimson (E_2)	$O_3 = \frac{1}{1+e^{-(w_{3A}\cdot A_T + w_{3H}\cdot A_H + b}}$
		FBNeu 23: X_1 (A_H), X_2 (A_T), X_3 (I_P); $w_{23H}(-)$, $w_{23A}(0)$, $w_{23I}(+)$; $y_{23} = w_{23H}A_H + w_{23A}A_T + w_{23I}I_P + b_{23}$ → O_3 (E_2)	BNeu 10: A_H, I_P; $w_{10H}(-)$, $w_{10I}(+)$; $y_{10} = w_{10H}A_H + w_{10I}I_P + b_{10}$ → E_G /E_2	E2-Crimson (E_2)	$O_{10} = \frac{1}{1+e^{-(w_{10I}\cdot I_P + w_{10H}\cdot A_H +}}$

Table S2: List of cellular devices constructed in this study.

Unit bactoneuro	Cellular device	Output cassette components	Regulatory cassette components

		Promoter –gene cassette	RBS	*Ori*	Antibiotic selection	Promoter-regulator cassette	RBS	*Ori*	Antibiotic selection
BNeu1	IAA1	P_{IAA}1-EGFP	R	pUC	Amp	-	-	-	-
	IAA2	P_{IAA}2-EGFP	R	pUC	Amp	-	-	-	-
	IAA3	P_{IAA}3-EGFP	R	pUC	Amp	-	-	-	-
	IAA4	P_{IAA}4-EGFP	R	pUC	Amp	-	-	-	-
	IAA5	P_{IAA}5-EGFP	R	pUC	Amp	-	-	-	-
	IAA6	P_{IAA}6-EGFP	R	pUC	Amp	-	-	-	-
	IAA7A	P_{IAA}7-EGFP	R	pUC	Amp	-	-	-	-
	IAA7B	P_{IAA}7-EGFP	R	p15A	Cm	-	-	-	-
	IAA8	P_{IAA}8-EGFP	R	pUC	Amp	-	-	-	-
	IAA9	P_{IAA}9-EGFP	R	pUC	Amp	-	-	-	-
	IAA10	P_{IAA}10-EGFP	R	pUC	Amp	-	-	-	-
	IAA11	P_{IAA}11-EGFP	R	pUC	Amp	-	-	-	-
	IAA7B.A	P_{IAA}7-E2-Crimson	R	p15A	Cm	-	-	-	-
	IAA7B.B	P_{IAA}7-mVenus	R	p15A	Cm	-	-	-	-
BNeu2	INA1A	P_{INA}1-EGFP	R	pUC	Amp	$P_{LtetO-1}$ -CI	R	p15A	Cm
	INA1B		R	pUC	Amp	$P_{LtetO-1}$ -CI	RC1	p15A	Cm
	INA1C		R	pUC	Amp	$P_{LtetO-1}$ -CI	RC2	p15A	Cm
	INA1D		R	pUC	Amp	$P_{LtetO-1}$ -CI	RC3	p15A	Cm
	INA2A	P_{INA}2-EGFP	R	pUC	Amp	$P_{LtetO-1}$ -CI	R	p15A	Cm
	INA2B		R	pUC	Amp	$P_{LtetO-1}$ -CI	RC1	p15A	Cm
	INA2C		R	pUC	Amp	$P_{LtetO-1}$ -CI	RC2	p15A	Cm
	INA2D		R	pUC	Amp	$P_{LtetO-1}$ -CI	RC3	p15A	Cm
	INA3A	P_{INA}3-EGFP	R	pUC	Amp	$P_{LtetO-1}$ -CI	R	p15A	Cm
	INA3B		R	pUC	Amp	$P_{LtetO-1}$ -CI	RC1	p15A	Cm
	INA3C		R	pUC	Amp	$P_{LtetO-1}$ -CI	RC2	p15A	Cm
	INA3D		R	pUC	Amp	$P_{LtetO-1}$ -CI	RC3	p15A	Cm
	INA4	P_{INA}4-EGFP	R	pUC	Amp	$P_{LtetO-1}$ -CI	R	p15A	Cm
	INA5A	P_{INA}5-EGFP	R	ColE1	Amp	$P_{LtetO-1}$ -CI	RC1	p15A	Cm
	INA5B		R	ColE1	Amp	$P_{LtetO-1}$ -CI	RC2	p15A	Cm
	INA5C		R	ColE1	Amp	$P_{LtetO-1}$ -CI	RC3	p15A	Cm
	INA6A	P_{INA}6-EGFP	R	ColE1	Amp	$P_{LtetO-1}$ -CI	RC1	p15A	Cm
	INA6B		R	ColE1	Amp	$P_{LtetO-1}$ -CI	RC2	p15A	Cm
	INA6C		R	ColE1	Amp	$P_{LtetO-1}$ -CI	RC3	p15A	Cm
	INA7A	P_{INA}7-EGFP	R	ColE1	Amp	$P_{LtetO-1}$ -CI	RC1	p15A	Cm
	INA7B		R	ColE1	Amp	$P_{LtetO-1}$ -CI	RC2	p15A	Cm
	INA7C		R	ColE1	Amp	$P_{LtetO-1}$ -CI	RC3	p15A	Cm
	INA7A.A	P_{INA}7-mTFP1	R	ColE1	Amp	$P_{LtetO-1}$ -CI	RC1	p15A	Cm
BNeu3	AAH1	P_{AAH}-EGFP	RBS H	p15A	Cm	BBa_J23102-LuxR	R	CoLE1	Amp
	AAH2	P_{AAH}-E2-Crimson	RBS H	p15A	Cm	BBa_J23102-LuxR	R	CoLE1	Amp
BNeu4	IAH1	P_{IAH}-EGFP	R	p15A	Cm	BBa_J23102-LuxR	R	CoLE1	Amp
	IAH2	P_{IAH}-tdTomato	R	p15A	Cm	BBa_J23102-LuxR	R	CoLE1	Amp
BNeu5	N1	P_R-EGFP	R	p15A	Cm	$P_{LlacO-1-}$ Frame-shifted CI* and $P_{LtaetO-1-}$ Frame-shifted CI*	R	ColE1	Amp
	N2	P_R-mKO2	R	p15A	Cm	$P_{LlacO-1-}$ Frame-shifted CI* and $P_{LtaetO-1-}$ Frame-shifted CI*	R	ColE1	Amp
BNeu 6	ANI1A	P_{ANI}1-EGFP	R	ColE1	Amp	$P_{LlacO-1}$ -CI	R	p15A	Cm
	ANI1B		R	ColE1	Amp	$P_{LlacO-1}$ -CI	RC1	p15A	Cm
	ANI1C		R	ColE1	Amp	$P_{LlacO-1}$ -CI	RC2	p15A	Cm
	ANI1D		R	ColE1	Amp	$P_{LlacO-1}$ -CI	RC3	p15A	Cm
	ANI2A	P_{ANI}2-EGFP	R	ColE1	Amp	$P_{LlacO-1}$ -CI	R	p15A	Cm
	ANI2B		R	ColE1	Amp	$P_{LlacO-1}$ -CI	RC1	p15A	Cm
	ANI2C		R	ColE1	Amp	$P_{LlacO-1}$ -CI	RC2	p15A	Cm
	ANI2D		R	ColE1	Amp	$P_{LlacO-1}$ -CI	RC3	p15A	Cm
	ANI3A	P_{ANI}3-EGFP	R	ColE1	Amp	$P_{LlacO-1}$ -CI	R	p15A	Cm
	ANI3B		R	ColE1	Amp	$P_{LlacO-1}$ -CI	RC1	p15A	Cm
	ANI3C		R	ColE1	Amp	$P_{LlacO-1}$ -CI	RC2	p15A	Cm
	ANI3D		R	ColE1	Amp	$P_{LlacO-1}$ -CI	RC3	p15A	Cm
	ANI4A	P_{ANI}4EGFP	R	ColE1	Amp	$P_{LlacO-1}$ -CI	R	p15A	Cm
	ANI4B		R	ColE1	Amp	$P_{LlacO-1}$ -CI	RC1	p15A	Cm
	ANI4C		R	ColE1	Amp	$P_{LlacO-1}$ -CI	RC2	p15A	Cm
	ANI4D		R	ColE1	Amp	$P_{LlacO-1}$ -CI	RC3	p15A	Cm

	ANI2C.A	P_{ANI}2-E2-Crimson	R	ColE1	Amp	$P_{LlacO-1}$ -CI	RC2	p15A	Cm
BNeu7	AHLB1	P_{Lux*}-EGFP	R	p15A	Cm	BBa_J23102-LuxR	R	CoLE1	Amp
	AHLB2	P_{Lux*}-E2-Crimson	R	p15A	Cm	BBa_J23102-LuxR	R	CoLE1	Amp
BNeu8	ATCB1	$P_{LtetO-1}$-E2-EGFP	R	ColE1	Amp	-	-	-	-
	ATCB2	$P_{LtetO-1}$-E2-Crimson	R	ColE1	Amp	-	-	-	-
BNeu 9	ANH1	P_{ANH}-EGFP	R	ColE1	Amp	BBa_J23102-LuxR	R	CoLE1	Amp
						P_{Lux}-Frame-shifted CI	R	p15A	Cm
	ANH2	P_{ANH}-tdTomato	R	ColE1	Amp	BBa_J23102-LuxR	R	CoLE1	Amp
						P_{Lux}-Frame-shifted CI	R	p15A	Cm
BNeu 10	INH1	P_{INH}-EGFP	R	ColE1	Amp	BBa_J23102-LuxR	R	CoLE1	Amp
						P_{Lux}-Frame-shifted CI	R	p15A	Cm
	INH2	P_{INH}-E2-Crimson	R	ColE1	Amp	BBa_J23102-LuxR	R	CoLE1	Amp
						P_{Lux}-Frame-shifted CI	R	p15A	Cm

*Frame-shifted CI is a mutant form of λ repressor CI [Supplementary reference 1].

Table S3: List of Promoters, primers, oligos and RBSs. *lac*O1, *tet*O2, Lux box, *O*R1 and *O*R2 operator sites are colored in red, brown, green, yellow and blue respectively. Transcription start site is shown in bold. -10 and -35 hexamers are underlined. Each promoter is flanked by *Xho*I and *EcoR*I restriction sites (marked in italics).

Name	Sequence (5' – 3')	Purpose	Source
P_{IAA}1	*CTCGAG*TCCCTATCAGTGATAGAGATTGACATTGTGAGCGGATAACAAGATACTGAGCAC**A**ATTGTGAGCGGATAACAAT*GAATTC*	Construction and weight & bias adjustment of BNeu 1	This study
P_{IAA}2	*CTCGAG*TCCCTATCAGTGATAGAGAGATTCCCTATCAGTGATAGAGATTGACATTGTGAGCGGATAACAAGATACTGAGCAC**A**ATTGTGAGCGGATAACAAT*GAATTC*		This study
P_{IAA}3	*CTCGAG*AATTGTGAGCGGATAACAATTGACATCCCTATCAGTGATAGAGATACTGAGCAC**A**TCCCTATCAGTGATAGAGA*GAATTC*		This study
P_{IAA}4	*CTCGAG*TCCCTATCAGTGATAGAGATTGACATTGTGAGCGGATAACAAGATACTGAGCAC**A**TCCCTATCAGTGATAGAGA*GAATTC*		This study
P_{IAA}5	CTCGAGTCCCTATCAGTGATAGAGATTGACATCCCTATCAGTGATAGAGATACTGAGCAC**A**ATTGTGAGCGGATAACAAT*GAATTC*		This study
P_{IAA}6	*CTCGAG*TCCCTATCAGTGATAGAGATTGACATTGTGAGCGGATAACAAGATACTGAGCAC**A**ATTGTGAGCGGATAACAATGATTCCCTATCAGTGATAGAGA*GAATTC*		This study
P_{IAA}7	*CTCGAG*TCCCTATCAGTGATAGAGATTGACATTGTGAGCGGATAACAAGATACTGAGCAC**A**TCCCTATCAGTGATAGAGAGATAATTGTGAGCGGATAACAATT*GAATTC*		This study
P_{IAA}8	*CTCGAG*TCCCTATCAGTGATAGAGATTGACATTGTGAGCGGATAACAAGATACTGAGCAC**A**TCCCTATCAGTGATAGAGAGATAATTGTGAGCGGATAACAATTGATAATTGTGAGCGGATAACAATT*GAATTC*		[Supplementary reference 1]
P_{IAA}9	*CTCGAG*TCCCTATCAGTGATAGAGATTGACATTGTGAGCGGATAACAAGATACTGAGCAC**A**TCCCTATCAGTGATAGAGAGATAATTGTGAGCGGATAACAATTGATTCCCTATCAGTGATAGAGA*GAATTC*		This study
P_{IAA}10	*CTCGAG*TCCCTATCAGTGATAGAGATTGACATTGTGAGCGGATAACAAGATACTGAGCAC**A**TCCCTATCAGTGATAGAGAGATGATAATTGTGAGCGGATAACAATTGATGATTCCCTATCAGTGATAGAGAGATGATAATTGTGAGCGGATAACAATT*GAATTC*		This study
P_{IAA}11	*CTCGAG*TCCCTATCAGTGATAGAGATTGACATTGTGAGCGGATAACAAGATACTGAGCAC**A**TCCCTATCAGTGATAGAGAGATGATAATTGTGAGCGGATAACAATTGATGATTCCCTATCAGTGATAGAGAGATGATAATTGTGAGCGGATAACAATTGATGATTCCCTATCAGTGATA		This study

	GAGAGATGATAATTGTGAGCGGATAACAATT*GAATTC*		
**P_{INA}1**	C*CTCGAG*TACCTCTGGCGGTGATATTGACATTGTGAGCGGATAACAAGATACTGAGCACAATTGTGAGCGGATAACAAT*GAATTC*	Construction and weight & bias adjustment of BNeu 2	This study
**P_{INA}2**	*CTCGAG*TACCTCTGGCGGTGATAGATTACCTCTGGCGGTGATATTGACATTGTGAGCGGATAACAAGATACTGAGCACAATTGTGAGCGGATAACAAT*GAATTC*		This study
**P_{INA}3**	*CTCGAG*TAACACCGTGCGTGTTGACTATTTTACCTCTGGCGGTGATAATGGTTGCAATTGTGAGCGGATAACAAT*GAATTC*		This study
**P_{INA}4**	*CTCGAG*TAACACCGTGCGTGTTGACTATTTTACCTCTGGCGGTGATAATGGTTGCAATTGTGAGCGGATAACAATGATAATTGTGAGCGGATAACAAT*GAATTC*		This study
**P_{INA}5**	*CTCGAG*AATTGTGAGCGGATAACAATTGACATTGTGAGCGGATAACAAGATACTGAGCACATCTACCTCTGGCGGTGATA*GAATTC*		This study
**P_{INA}6**	*CTCGAG*AATTGTGAGCGGATAACAATTGACATTGTGAGCGGATAACAAGATACTGAGCACATCTACCTCTGGCGGTGATAGATGATTACCTCTGGCGGTGATA*GAATTC*		This study
**P_{INA}7**	*CTCGAG*AATTGTGAGCGGATAACAATTGACATTGTGAGCGGATAACAAGATACTGAGCACATCTACCTCTGGCGGTGATAGATGATTACCTCTGGCGGTGATAGATGATTACCTCTGGCGGTGATA*GAATTC*		This study
**P_{ANI}1**	*CTCGAG*TACCTCTGGCGGTGATATTGACATCCCTATCAGTGATAGAGATACTGAGCACATCCCTATCAGTGATAGAGA*GAATTC*	Construction and weight & bias adjustment of BNeu 6	This study
**P_{ANI}2**	C*CTCGAG*TACCTCTGGCGGTGATAGATTACCTCTGGCGGTGATATTGACATCCCTATCAGTGATAGAGATACTGAGCACATCCCTATCAGTGATAGAGA*GAATTC*		This study
**P_{ANI}3**	*CTCGAG*TACCTCTGGCGGTGATATTGACATCCCTATCAGTGATAGAGATACTGAGCACATCTACCTCTGGCGGTGATA*GAATTC*		This study
**P_{ANI}4**	*CTCGAG*TCCCTATCAGTGATAGAGATTGACATACCTCTGGCGGTGATAGATACTGAGCACATCCCTATCAGTGATAGAGA*GAATTC*		This study
**P_{AAH}**	*CTCGAG*ACCTGTAGGATCGTACAGGTTTACGTCCCTATCAGTGATAGAGTATAGTCGAATAAATCCCTATCAGTGATAGAGA*GAATTC*	Construction of BNeu 3	This study
**P_{IAH}**	*CTCGAG*ACCTGTAGGATCGTACAGGTTTACGTTTGTGAGCGGATAACAATATAGTCGAATAAATTGTGAGCGGATAACAATT*GAATTC*	Construction of BNeu 4	This study
**P_R**	*CTCGAG*TAACACCGTGCGTGTTGACTATTTTACCTCTGGCGGTGATAATGGTTGCATGTAC*GAATTC*	Construction of BNeu 5	[1]
**BBa_J23102**	*CTCGAG*TTGACAGCTAGCTCAGTCCTAGGTACTGTGCTAGC*GAATTC*	Construction of BNeus 3, 4, 7, 9 and 10	[2]
**P_{Lux}**	*CTCGAG*ACCTGTAGGATCGTACAGGTTTACGCAAGAAAATGGTTTGTTATAGTCGAATAAA*GAATTC*	Construction of BNeus 9 and 10	This study
**P_{Lux}***	*CTCGAG*ACCTGTAGGATCGTACAGGTTTACGCAAGAAAATGGTTTGTTACTTTCGAATAAA*GAATTC*	Construction of BNeu 7	[3]
**$P_{LtetO-1}$**	*CTCGAG*TCCCTATCAGTGATAGAGATTGACATCCCTATCAGTGATAGAGATACTGAGCACATCAGCAGGACGCACTGACC*GAATTC*	Construction of BNeus 5 and 8	[4]
**$P_{LlacO-1}$**	*CTCGAG*AATTGTGAGCGGATAACAATTGACATTGTGAGCGGATAACAAGATACTGAGCACATCAGCAGGACGCACTGACC*GAATTC*	Construction of BNeu 5	[4]
**Primer 1**	GCCCTTTCGTCTTCACCTC	Amplification of promoters P_{IAA}1, P_{IAA}2, P_{INA}1, P_{INA}2, P_{ANI}1, P_{ANI}2, P_{ANI}3 and P_{ANI}4: forward primer	This study
**Primer 2**	ATGTTTTTGGCGTCTTCCAT	Amplification of promoters P_{IAA}1, P_{IAA}2, P_{INA}1, P_{INA}2, P_{ANI}1, P_{ANI}2, P_{ANI}3 and P_{ANI}4: reverse primer	This study

**Primer 3**	CGAGGCCCTTTCGTCTTCACCTCGAGAATTG TGAGCGGATAACAATTGACATCCCTATCAG TGATAGAGATACTGAGCACA	Amplification of promoter P_{IAA}3: forward primer	This study
**Primer 4**	ATGTTTTTGGCGTCTTCCATGGTACCTTTCT CCTCTTTAATGAATTCTCTCTATCACTGATA GGGATGTGCTCAGTATCTCTATCA	Amplification of promoter P_{IAA}3: reverse primer	This study
**Primer 5**	CGAGGCCCTTTCGTCTTCACCTCGAGTCCCT ATCAGTGATAGAGATTGACATTGTGAGCGG ATAACAAGATACTGAGCACATC	Amplification of promoter P_{IAA}4: forward primer	This study
**Primer 6**	ATGTTTTTGGCGTCTTCCATGGTACCTTTCT CCTCTTTAATGAATTCTCTCTATCACTGATA GGGATGTGCTCAGTATCTTGTT	Amplification of promoter P_{IAA}4: reverse primer	This study
**Primer 7**	CAATTCTTTATGCCGGTGTTG	Amplification of promoters P_{IAA}5 and P_{IAA}10: forward primer	This study
**Primer 8**	GTCGAAGATGTTGGGGTGTT	Amplification of promoters P_{IAA}5 and P_{IAA}10: reverse primer	This study
**Primer 9**	CAGAATCGTCGTATGCAGTGA	Amplification of promoters P_{IAA}6 and P_{IAA}11: forward primer	This study
**Primer 10**	TTTTCCGTCATCGTCTTTCC	Amplification of promoters P_{IAA}6 and P_{IAA}11: reverse primer	This study
**Primer 11**	TTGGCAGAAGCTATGAAACGA	Amplification of promoters P_{IAA}7, P_{INA}5 and P_{AAH}: forward primer	This study
**Primer 12**	CTTGACTGGCGACGTAATCC	Amplification of promoters P_{IAA}7, P_{INA}5 and P_{AAH}: reverse primer	This study
**Primer 13**	GCCCTTTCGTCTTCACCTC	Amplification of promoters P_{IAA}8 and P_{IAA}9: forward primer	This study
**Primer 14**	CTTGACTGGAATTCAATTGTTATCCGCTCAC AATTATCAATTGTTATCCGCTCACAATTATC TCTCTATCACTGATAGGGATGTGCTCAG	Amplification of promoter P_{IAA}8: reverse primer	This study
**Primer 15**	CTTGACTGGAATTCTCTCTATCACTGATAGG GAATCAATTGTTATCCGCTCACAATTATCTC TCTATCACTGATAGGGATGTGCTCAG	Amplification of promoter P_{IAA}9: reverse primer	This study
**Primer 16**	CAGATGCACATATCGAGGTGA	Amplification of promoters P_{INA}3 and P_{INA}6: forward primer	This study
**Primer 17**	GCAACTTTTTGGCGGTTG	Amplification of promoters P_{INA}3 and P_{INA}6: reverse primer	This study
**Primer 20**	TGAAGAGATACGCCCTGGTT	Amplification of promoter P_{INA}4, P_{INA}7 and P_{IAH}: forward primer	This study
**Primer 21**	TCTGATTTTTCTTGCGTCGAG	Amplification of promoter P_{INA}4, P_{INA}7 and P_{IAH}: reverse primer	This study
**Primer 22**	ATCCGTGCAACTCGAGTTGACAGCTAGCTC AGTCCTAGGTAC	Amplification of promoter BBa_J23102: forward primer	This study
**Primer 23**	GTTCAAGACTGAATTCGCTAGCACAGTACC TAGGACTGAGCTAGC	Amplification of promoter BBa_J23102: reverse primer	This study
**Primer 24**	CTTCACTCGACTCGAGACCTGTAGGATCGT ACAGGTTTACGCAAGAAAATGG	Amplification of promoters P_{Lux} and P_{Lux}*: forward primer	This study
**Primer 25**	CTGATTATGTGAATTCTTTATTCGAAAGTAA CAAACCATTTTCTTGCGTAAACCTG	Amplification of promoter P_{Lux}*: reverse primer	This study
**Primer 26**	GAGACCACAATGGGCGTAAT	Amplification of fluorescent protein mTFP1: forward primer (1st round)	This study
**Primer 27**	CGTAAACGGTCACCTTGTTGTA	Amplification of fluorescent protein mTFP1: reverse primer (1st round)	This study
**Primer 28**	GTCCAGTCGAGGTACCATGGTGAGCAAGGG CGAGGAGACCACAATGGGCGTAAT	Amplification of fluorescent protein mTFP1: forward primer (2nd round)	This study
**Primer 29**	GCTTATGCTCTAGATTACTTGTACAGCTCGT CCATGCCGTCGGTGGAGTTGCGGCCACGG CGCTCTCGTAAACGGTCACCTTGTTGTA	Amplification of fluorescent protein mTFP1: reverse primer (2nd round)	This study
**Primer 30**	CAAGGGCGAGGAGCTGTT	Amplification of fluorescent protein EGFP and mVenus: forward primer (1st round)	This study
**Primer 31**	CCATGCCGAGAGTGATCC	Amplification of fluorescent protein EGFP and mVenus: reverse primer (1st round)	This study
**Primer 32**	CTTCAGTCGAGGTACCATGGTGAGCAAGGG CGAGGAGCTGTT	Amplification of fluorescent protein EGFP and mVenus: forward primer (2nd round)	This study
**Primer 33**	CTGATTATGATCTAGATTACTTGTACAGCTC GTCCATGCCGAGAGTGATCC	Amplification of fluorescent protein EGFP and mVenus: reverse primer (2nd round)	This study

**Primer 34**	TGGTGAGTGTGATTAAACCAGAGA	Amplification of fluorescent protein mKO2: forward primer (1st round)	This study
**Primer 35**	AATGTTGCCTTCGGTTTTCC	Amplification of fluorescent protein mKO2: reverse primer (1st round)	This study
**Primer 36**	GTCCAGTCGAGGTACCATGGTGAGTGTGATTAAACCAGAGA	Amplification of fluorescent protein mKO2: forward primer (2nd round)	This study
**Primer 37**	GTGATTATGATCTAGATTAGCTATGAGCTACTGCATCTTCTACCTGCTCAGTAATGTTGCCTTCGGTTTTCC	Amplification of fluorescent protein mKO2: reverse primer (2nd round)	This study
**Primer 38**	TGGATAGCACTGAGAACGTCAT	Amplification of fluorescent protein E2-Crimson: forward primer (1st round)	This study
**Primer 39**	ACCACGGTGTAGTCCTCGTT	Amplification of fluorescent protein E2-Crimson: reverse primer (1st round)	This study
**Primer 40**	GTCCAGTCGAGGTACCATGGATAGCACTGAGAACGTCAT	Amplification of fluorescent protein E2-Crimson: forward primer (2nd round)	This study
**Primer 41**	GATTATGATCTAGActaCTGGAACAGGTGGTGGCGGGCCTCGGCGCGCTCGTACTGCTCCACCACGGTGTAGTCCTCGTT	Amplification of fluorescent protein E2-Crimson: reverse primer (2nd round)	This study
**Primer 42**	ATGCCGACGACACATACAGA	Amplification of LuxR gene: forward primer (1st round)	This study
**Primer 43**	TGATGCCTGGCTCTAGTAGTGA	Amplification of LuxR gene: reverse primer (1st round)	This study
**Primer 44**	CTCCGTGGAAGGTACCATGAAAAACATAAATGCCGACGACACATACAGA	Amplification of LuxR gene: forward primer (2nd round)	This study
**Primer 45**	GTTCAAGACTTCTAGATGATGCCTGGCTCTAGTAGTGA	Amplification of LuxR gene: reverse primer (2nd round)	This study
**Primer 46**	CGAAAAGTGCCACCTGAC	Amplification of gene cassette starting with promoters $P_{LtetO-1}$, P_{IAA}1-2, P_{IAA}4-11 and P_{ANI}4: forward sequencing primer	This study
**Primer 47**	GTCTGATTGAGAATTCATTTTTGAGGAGTTCGGTACCATGGTGAGCAAGGGCGAGGAGCTGTT	Incorporation of RC1 upstream of EGFP gene: forward primer	This study
**Primer 48**	GTCTGATTGAGAATTCATTCGGGAGGAGTGCGGTACCATGGTGAGCAAGGGCGAGGAGCTGTT	Incorporation of RC2 upstream of EGFP gene: forward primer	This study
**Primer 49**	GTCTGATTGAGAATTCATTTCGGAGGAGTGCGGTACCATGGTGAGCAAGGGCGAGGAGCTGTT	Incorporation of RC3 upstream of EGFP gene: forward primer	This study
**Primer 50**	CTGATTATGTGAATTCTTTATTCGACTATAACAAACCATTTTCTTGCGTAAACCTG	Amplification of promoter P_{Lux}: reverse primer	This study
**Oligo 1**	AATTCATTGGAGAGGAGTCCGGTAC	RBSH: sense strand oligomer for annealing	This study
**Oligo 2**	CGGACTCCTCTCCAATG	RBSH: antisense strand oligomer for annealing	This study

Table S4: Weights and biases of each cellular device (construct) used for optimizing and improving corresponding unit bactoneuron (BNeu j).

Unit bactoneuron	Cellular device	w_{jR}	w_{jI}	w_{jA}	w_{jH}	b_{jR}	b_{jI}	b_{jA}	b_{jH}	b_j	S.D. of b_j
BNeu1	IAA1	-	7.75	8.42	-	-	-1.26	-2.15	-	-9.79	0.16
	IAA2	-	10.37	9.05	-	-	-1.68	-0.77	-	-10.94	0.29
	IAA3	-	8.94	15.44	-	-	0.67	-5	-	-14.36	0.59
	IAA4	-	6.19	11.77	-	-	0.18	-5.12	-	11.45	0.198
	IAA5	-	7.71	12.6	-	-	-1.09	-6.02	-	-13.71	0.03
	IAA6	-	7.2	7.07	-	-	-0.81	-0.64	-	-7.86	0.03
	IAA7A	-	8.5	11.73	-	-	-1.73	-4.52	-	-13.24	0.31
	IAA7B(10h+6h)	-	9.69	12.44	-	-	-2.58	-5.59	-	-15.15	0.18
	IAA8	-	8.36	11.92	-	-	-1.47	-4.8	-	-13.28	0.16
	IAA9	-	7.58	12.96	-	-	-1.15	-6.27	-	-13.98	0.18
	IAA10	-	8.84	15.91	-	-	-1.41	-8.26	-	-17.21	0.16
	IAA11	-	9.94	12.44	-	-	-1.71	-3.55	-	-13.82	0.47
	IAA7B.A	-	9.69	12.44	-	-	-2.58	-5.59	-	-15.15	0.18
	IAA7B.B	-	9.69	12.44	-	-	-2.58	-5.59	-	-15.15	0.18
BNeu2	INA6A	-	9.59	-15.32	-	-	-2.49	7	-	-2.54	0.07
	INA6B	-	9.11	-14.87	-	-	-2.12	7	-	-2.12	0.007

	INA6C	-	9.12	-15.48	-	-	-1.71	7.5	-	-1.67	0.07
	INA7A	-	10.98	-14.57	-	-	-2.68	7.8	-	-2.93	0.35
	INA7A (10h+6h)	-	10.8	-14.89	-	-	-3.3	7.14	-	-3.48	0.25
	INA7B	-	9.51	-14.89	-	-	-2.12	7.5	-	-2.07	0.08
	INA7A.A	-	10.8	-14.89	-	-	-3.3	7.14	-	-3.48	0.25
BNeu3	AAH1(10h+6h)	-	0	13.16	10.93	-	-	-4.52	-2.42	-15.52	0.09
	AAH2	-	0	13.16	10.93	-	-	-4.52	-2.42	-15.52	0.09
BNeu4	IAH1(10h+6h)	-	10.78	0	11.98	-	-2.12	-	-3.17	-14.03	0.11
	IAH2	-	10.78	0	11.98	-	-2.12	-	-3.17	-14.03	0.11
BNeu5	N1(10h+6h)	-	-10.03	-11.65	-	-	3.52	3.72	-	3.62	0.14
	N2	-	-10.03	-11.65	-	-	3.52	3.72	-	3.62	0.14
BNeu6	ANI2C	-	-15.58	12.59	-	-	7.5	-4.3	-	-4.7	0.56
	ANI2C (10h+6h)		-16.25	13.2	-	-	7.5	-4.64		-5.17	0.75
	ANI2C.A	-	-16.25	13.2	-	-	7.5	-4.64	-	-5.17	0.75
BNeu7	AHLB1(10h+6h)	0	0	0	10.84	-	-	-	-3.21	-3.21	-
	AHLB2	0	0	0	10.84	-	-	-	-3.21	-3.21	-
BNeu8	ATCB1(10h+6h)	0	0	12.17	0	-	-	-4.85	-	-4.85	-
	ATCB2	0	0	12.17	0	-	-	-4.85	-	-4.85	-
BNeu 9	ANH1(10h+6h)	-	0	10.00	-12.94	-	-	-3.2	5.8	-3.7	0.71
	ANH2	-	0	10.00	-12.94	-	-	-3.2	5.8	-3.7	0.71
BNeu 10	INH1(10h+6h)	-	9.42	0	-13.75	-	-1.83	-	7.00	-2.13	0.42
	INH2	-	9.42	0	-13.75	-	-1.83	-	7.00	-2.13	0.42

Table S5: Leakage of each EGFP-expressing cellular device (construct) during weight and bias optimization of unit bactoneurons.

Unit bactoneuron	Cellular device	Promoter expressing Output EGFP	Total leakage (∑L)	Highest leakage (L_{max})	Percentage highest leakage (L_{max}(%))	Difference between total leakage and highest leakage (∑L- L_{max})	Percentage difference between total leakage and highest leakage (∑L- L_{max} (%))	Fold Change between highest signal and highest leakage
BNeu 1	IAA1	$P_{IAA}1$	0.44075	0.27818	27.82	0.16257	16.26	3.59
	IAA2	$P_{IAA}2$	0.38592	0.26795	26.8	0.11797	11.8	3.73
	IAA3	$P_{IAA}3$	0.26577	0.23858	23.86	0.02719	2.72	4.19
	IAA4	$P_{IAA}4$	0.2465	0.23556	23.56	0.01094	1.09	4.25
	IAA5	$P_{IAA}5$	0.22367	0.17495	17.5	0.04872	4.87	5.72
	IAA6	$P_{IAA}6$	0.42029	0.26436	26.44	0.15593	15.59	3.78
	IAA7A	$P_{IAA}7$	0.12599	0.11025	11.03	0.01574	1.57	9.07
	IAA7B		0.0724	0.06992	6.99	0.00248	0.25	14.3
	IAA8	$P_{IAA}8$	0.11287	0.09674	9.67	0.01613	1.61	10.33
	IAA9	$P_{IAA}9$	0.15845	0.13822	13.82	0.02023	2.02	7.23
	IAA10	$P_{IAA}10$	0.23014	0.21021	21.02	0.01993	1.99	4.76
	IAA11	$P_{IAA}11$	0.52619	0.49162	49.16	0.03457	3.46	2.03
BNeu 2	INA1A	$P_{INA}1$	0.63673	0.28273	28.27	0.354	35.4	3.54
	INA1B		0.53862	0.36044	36.04	0.17819	17.82	2.77
	INA1C		0.74802	0.45623	45.62	0.29179	29.18	2.19
	INA1D		0.80989	0.45047	45.05	0.35942	35.94	2.22
	INA2A	$P_{INA}2$	0.72654	0.37157	37.16	0.35497	35.5	2.69
	INA2B		0.63688	0.53902	53.9	0.09787	9.79	1.86
	INA2C		0.79001	0.51294	51.29	0.27707	27.71	1.95
	INA2D		0.85405	0.4802	48.02	0.37385	37.39	2.08
	INA3A	$P_{INA}3$	0.77393	0.38146	38.15	0.39247	39.25	2.62
	INA3B		0.72405	0.70337	70.34	0.02067	2.07	1.42
	INA3C		0.89997	0.80164	80.16	0.09832	9.83	1.25
	INA3D		2.13488	0.8703	87.03	1.26457	126.46	1.15
	INA4	$P_{INA}4$	1.23277	0.80884	80.88	0.42393	42.39	1.24
	INA5A	$P_{INA}5$	0.40655	0.23903	23.9	0.16752	16.75	4.18
	INA5B		0.35622	0.23112	23.11	0.1251	12.51	4.33
	INA5C		0.56242	0.38104	38.1	0.18138	18.14	2.62
	INA6A	$P_{INA}6$	0.10504	0.06611	6.61	0.03893	3.89	15.13
	INA6B		0.0933	0.06478	6.48	0.02852	2.85	15.44

	INA6C		0.16935	0.08823	8.82	0.08112	8.11	11.33
	INA7A	$P_{INA}7$	0.07783	0.04258	4.26	0.03525	3.53	23.49
	INA7B		0.09163	0.04595	4.6	0.04567	4.57	21.76
	INA7C		0.40418	0.34627	34.63	0.05791	5.79	2.89
BNeu 3	AAH1	P_{AAH}	0.05393	0.0502	5.02	0.00373	0.37	19.92
BNeu 4	IAH1	P_{IAH}	0.1402	0.08861	8.86	0.05159	5.16	11.29
BNeu 5	N1	P_R	~0.00000	~0.00000	~00.00	~0.00000	~00.00	-
BNeu 6	ANI1A	$P_{ANI}1$	0.90311	0.74394	74.39	0.15917	15.92	1.34
	ANI1B		0.20376	0.17735	17.73	0.02642	2.64	5.64
	ANI1C		0.2262	0.20363	20.36	0.02256	2.26	4.91
	ANI1D		0.63651	0.62022	62.02	0.01629	1.63	1.61
	ANI2A	$P_{ANI}2$	0.60315	0.40034	40.03	0.20281	20.28	2.5
	ANI2B		0.22933	0.17476	17.48	0.05457	5.46	5.72
	ANI2C		0.15726	0.10908	10.91	0.04818	4.82	9.16
	ANI2D		0.26405	0.23224	23.22	0.03181	3.18	4.31
	ANI3A	$P_{ANI}3$	3.1181	2.43408	243.41	0.68398	68.4	0.41
	ANI3B		0.51524	0.3359	33.59	0.17933	17.93	2.98
	ANI3C		0.44074	0.22444	22.44	0.21629	21.63	4.46
	ANI3D		0.40381	0.30815	30.82	0.09566	9.57	3.25
	ANI4A	$P_{ANI}4$	0.7652	0.47934	47.93	0.28586	28.59	2.09
	ANI4B		0.18267	0.16603	16.6	0.01664	1.66	6.02
	ANI4C		0.25445	0.23556	23.56	0.0189	1.89	4.25
	ANI4D		0.60619	0.59089	59.09	0.0153	1.53	1.69
BNeu 7	AHLB1	P_{Lux} *	0.0338	0.0338	3.38	-	-	29.59
BNeu 8	ATCB1	$P_{LtetO-1}$	0.00778	0.00778	0.78	-	-	128.53
BNeu 9	ANH1	$P_{ANI}2$	0.13249	0.08548	8.55	0.04701	4.7	11.7
BNeu 10	INH1	$P_{INA}7$	0.17488	0.09012	9.01	0.08476	8.48	11.1

Table S6: Translation initiation rate calculated from RBS calculator [5].

Name of RBS	Operating Promoter	Protein of Translational regulation	Translation initiation rate (a.u.)	Sequence (5' – 3')
R(BBa_B0034) [6]	$P_{LlacO-1}$ /$P_{LtetO-1}$	CI	40767	GAATTCATTAAAGAGGAGAAAGGTACC
RC1	$P_{LlacO-1}$ /$P_{LtetO-1}$	CI	2091	GAATTCATTTTTGAGGAGTTCGGTACC
RC2	$P_{LlacO-1}$ /$P_{LtetO-1}$	CI	518	GAATTCATTCGGGAGGAGTGCGGTACC
RC3	$P_{LlacO-1}$ /$P_{LtetO-1}$	CI	396	GAATTCATTTCGGAGGAGTGCGGTACC
R(BBa_B0034) [6]	P_{AAH}	EGFP	46	GAATTCATTAAAGAGGAGAAAGGTACC
RH	P_{AAH}	EGFP	10	GAATTCATTGGAGAGGAGTCCGGTACC

Table S7: Details of molecular engineering performed for weight and bias optimization of the unit bactoneurons.

Unit bactoneuron	Molecular engineering of the cellular devices for weight and bias optimization
BNeu 1	**Initial assumptions:** We took the design knowledge from two reported synthetic promoters showing nonlinear behavior with respect to IPTG and aTc [4] to make a starting set (Set 1) of five synthetic promoters $P_{IAA}1$-5 carried by cellular devices IAA1-5. The designs of those synthetic promoters were made by varying the number and relative positions of the operator sites for LacI and TetR. Schematic representation of the promoter maps can be found in figure 3 and the promoter sequences are shown in supplementary table S3.<br><br>**Initial characterization of cellular devices IAA1-5: (Single-cassette systems in high copy (pUC ori) plasmids)** •No device was found showing ≥8 fold between highest signal and highest leakage (Table S5) •Either weight values were low, or the difference between IPTG weight and aTc weight was high (Table S4)<br><br>In most cases, IPTG weight had lower value than aTc weight (Table S4). The weight values represent the slope in the dose response curve (See equation 1 in the main text). Among those five devices, total leakage for IAA4 and IAA5 was similar (Table S5). However, although IAA5 showed lowest value of highest leakage, IAA4 showed minimum leakage associated with the input states other than the highest leakage state (Difference between $\sum L$ and $\sum L_{max}$). Therefore, we selected IAA4 as the design template for the next set of cellular devices (Set 2) in order to decrease the leakage and sharpen the slope (weight value) of the bactoneural response curve with respect to the input inducers which was the optimization of the weights of the inputs.

	Weight and leakage adjustment step 1 to decrease the leakage and sharpen the slope of the bactoneural response curve<br><br>The aTc single induction state was the highest leakage state of IAA4 (Figure 3, figure S2 and table S5). Thus, we assumed that, the device produced leakage due to insufficient interactions between LacI transcription factor and its operator sites present in the promoter $P_{IAA}4$ which was supposed to remain turned off because of the LacI binding in aTc single induction condition. Based on this assumption, we increased the number of binding sites with different combinations and relative positions to generate two more promoters $P_{IAA}6$ and 7 carried by cellular devices IAA6 and IAA7A respectively (Figure 3, table S2 and table S3). Increased number of operator sites would promote more LacI binding events causing tight repression of the promoter and therefore leakage reduction. It was previously reported that, the slope of the circuit response curve with respect to the input could be altered by changing the number and the relative positions of the transcription factor-specific operator sites in the target promoter design [7]**.** Here we thought that the same could be applicable to make the device more sensitive to the input inducers that in turn could sharpen the bactoneural response curve leading to the elevated weight values.<br><br>**Initial characterization of cellular devices IAA6 and IAA7A:** •More than 8 fold between highest signal and highest leakage was achieved only for IAA7A (Table S5)<br>•Value of IPTG weight was increased for IAA7A (Table S4)<br><br>Hence, we selected IAA7A as the best design form Set 2. We thought to further optimize weights and bias for this bactoneuron (Set 3).<br><br>Weight and leakage adjustment step 2<br><br>We again increased the operator sites and designed promoters $P_{IAA}8$-11 carried by cellular devices IAA8-11 respectively (Figure 3, table S2 and table S3).<br>**Initial characterization of cellular devices IAA8-11:** •Leakage built up gradually (Table S5)<br><br>No device was selected. We stopped increasing the number of operator sites on the synthetic promoters.<br><br>We changed the copy number of the plasmid carrying cellular device IAA7A from high copy (pUC) to low copy (p15A) to alter the relative amount of the promoter and its transcription factor per cell resulting in IAA7B (Figure 3 and table S2).<br>**Initial characterization of cellular device IAA7B:** •Highest fold change between highest signal and highest leakage (Table S5)<br>• Lowest leakage (Table S4)<br><br>Selected as the final cellular device<br><br>Cellular devices IAA7B.A and IAA7B.B: Developed from IAA7B by changing the output from EGFP to E2-Crimson and mVenus respectively (Table S2) only for microscopic experiments.
BNeu 2	**Initial assumptions:** Based on the design knowledge of BNeu 1 and the map of the P_R-P_{RM} promoter system of λ phage regulated by CI, four initial designs of the output-expressing synthetic promoters $P_{INA}1$-4 were created by varying number and relative positions of operator sites for LacI and CI (Figure 3 and table S3). They were placed under low copy origin (p15A) while their regulator CI was placed under high copy origin (pUC). CI shows high basal level expression (CI expression from inducible promoter even in absence of the corresponding input inducer) under a strong RBS [Supplementary reference 1] that would affect the desired bactoneural behavior. Therefore, to reduce its basal level expression through reduction of its translation rate, three weak RBSs RC1-3 were designed (Table S3). Individual weak RBSs along with the native strong RBS R (BBa_B0034) were co-transformed with each of the $P_{INA}1$-3 promoters, whereas, for $P_{INA}4$ promoter, only RBS R was tested (Table S2). As a result, in set 1, 13 cellular devices were built (INA1A-D, INA2A-D, INA3A-D and INA4).<br><br>**Initial characterization of cellular devices INA1A-4: (Double-cassette systems)** •Fold change between highest signal and highest leakage was very low (~1.1-3.5 fold) for all designs (Table S5)<br>•High leakage accumulation was observed in all cases (Table S5)<br><br>We didn't choose any devise from set 1 as no one fulfilled our first selection criterion that is at least 8 fold changes between the highest signal and the highest leakage. We decided to create completely new promoter designs.

	↓ Weight and leakage adjustment step 1<br><br>Next, we made another set (Set 2) of synthetic promoters P_{INA}5-7 (Table S3) by varying the number and relative positions of the LacI and CI binding sites. Here also, we placed CI under weak RBSs RC1-3 but we didn't consider strong RBS R anymore (Figure 3). We also changed the plasmid copy number for CI from high copy (pUC ori) to medium copy (ColE1 ori). In this way 9 more device designs were generated (INA5A-C, INA6A-C and INA7A-C).<br><br>**Initial characterization of cellular devices INA5A-7C:** •INA7A showed highest fold change between highest signal and highest leakage (~23.5 fold; Table S5)<br>•INA7A showed good weight values compared to cellular device IAA7B for BNeu 1 (Table S4)<br><br>↓<br><br>INA7A was selected as the final cellular device<br><br>Cellular device INA7A.A: Developed from INA7A by changing the output from EGFP to mTFP1 (Table S2) only for microscopic experiments.
BNeu 3	**Initial assumptions:** Based on the design knowledge of BNeu 1, the map of the bacterial P_{LUX} promoter (Table S3) regulated by LuxR, and the design of a reported synthetic promoter-based system showing nonlinear behavior with respect to IPTG, aTc and AHL [2], the design of the output-expressing synthetic promoter P_{AAH} was created (Figure S4 and table S3).<br><br>**Initial characterization of cellular device AAH1:** •Total leakage was low (Table S5)<br>**(Double-cassette system)** •Difference between total leakage and highest leakage was Low (Table S5)<br>•Fold change between highest signal and highest leakage was more than 8 fold (Table S5)<br>• Good weight values as compared to other bactoneurons (Table S4)<br><br>↓<br><br>Selected as the final cellular device<br><br>Cellular device AAH2: Developed from AAH1 by changing the output from EGFP to E2-Crimson (Table S2) only for microscopic experiments.
BNeu 4	**Initial assumptions:** Based on the design knowledge of BNeu 1 and BNeu 2, the map of the bacterial P_{LUX} promoter {Table S3) regulated by LuxR, and the design of a reported synthetic promoter-based system showing nonlinear behavior with respect to IPTG, aTc and AHL [2], the design of the output-expressing synthetic promoter P_{IAH} was created (Figure S4 and table S3).<br><br>**Initial characterization of cellular device IAH1:** •Total leakage was low (Table S5)<br>**(Double-cassette system)** •Difference between total leakage and highest leakage was Low (Table S5)<br>•Fold change between highest signal and highest leakage was more than 8 fold (Table S5)<br>•Good weight values as compared to other bactoneurons (Table S4)<br><br>↓<br><br>Selected as the final cellular device<br><br>Cellular device IAH2: Developed from IAH1 by changing the output from EGFP to tdTomato (Table S2) only for microscopic experiments.
BNeu 5	**Initial assumptions:** Based on a biological NOT gate, developed previously [1], cellular device N1 was designed.<br><br>**Initial characterization of cellular device N1:** •Total leakage was low (Table S5)<br>**(Double-cassette system)** •Difference between total leakage and highest leakage was low (Table S5)<br>•Fold change between highest signal and highest leakage was more than 8 fold (Table S5)<br>•Good weight values as compared to other bactoneurons (Table S4)

	Selected as the final cellular device<br><br>Cellular device N2: Developed from N1 by changing the output from EGFP to mKO2 (Table S2) only for microscopic experiments.
BNeu 6	**Initial assumptions:** Based on the design knowledge of BNeu 1 and BNeu 2, four initial designs of the output-expressing synthetic promoters P_{ANI}1-4 were made by varying number and relative positions of operator sites for TetR and CI (Figure 3 and table S3). They were placed under low copy origin (p15A) while their regulator CI was placed under medium copy origin (ColE1). Similar to the bactoneuron BNeu 2, weak RBSs RC1-3 along with the native RBS R (BBa_B0034) were co-transformed with each of the P_{ANI}1-4 promoters resulting in a set (Set 1) of 12 designs (ANI1A-D, ANI2A-D, ANI3A-D and ANI4A-D).<br><br>**Initial characterization of cellular devices ANI1A-4D: (Double-cassette systems)**<br>•Only ANI2C showed more than 8 fold change between highest signal and highest leakage (Table S5)<br>• ANI2C showed good weight values compared to other bactoneurons (Table S4)<br><br>ANI2C was selected as the final cellular device<br><br>Cellular device ANI2C.A: Developed from ANI2C by changing the output from EGFP to E2-Crimson (Table S2) only for microscopic experiments.
BNeu 7	**Initial assumptions:** Based on the design knowledge of BNeu 3 and BNeu 4, cellular device AHLB1 was designed (Figure S4 and table S2).<br><br>**Initial characterization of cellular device AHLB1: (Double-cassette system)**<br>•Total leakage was low (Table S5)<br>•Difference between total leakage and highest leakage was Low (Table S5)<br>•Fold change between highest signal and highest leakage was more than 8 fold (Table S5)<br>•Good weight value as compared to other bactoneurons (Table S4)<br><br>Selected as the final cellular device<br><br>Cellular device AHLB2: Developed from AHLB1 by changing the output from EGFP to E2-Crimson (Table S2) only for microscopic experiments.
BNeu 8	**Initial assumptions:** Based on the design knowledge of the reported synthetic promoter $P_{LtetO-1}$ showing nonlinear behavior with respect to aTc [4], cellular device ATCB1 was designed (Figure S4 and table S2).<br><br>**Initial characterization of cellular device ATCB1: (Single-cassette system in medium copy (ColE1 ori) plasmid)**<br>•Total leakage was low (Table S5)<br>•Difference between total leakage and highest leakage was low (Table S5)<br>• Fold change between highest signal and highest leakage was more than 8 fold (Table S5)<br>• Good weight value as compared to other bactoneurons (Table S4)<br><br>Selected as the final cellular device<br><br>Cellular device ATCB2: Developed from ATCB1 by changing the output from EGFP to E2-Crimson (Table S2) only for microscopic experiments.
BNeu 9	**Initial assumptions:** Based on the design knowledge of BNeu 3, BNeu 6 and BNeu 7, cellular device ANH1 was designed (Figure S4 and table S2).<br><br>**Initial characterization of cellular device ANH1: (Double-cassette system)**<br>•Total leakage was low (Table S5)<br>•Difference between total leakage and highest leakage was Low (Table S5)<br>• Fold change between highest signal and highest leakage

	was more than 8 fold(Table S5)<br><br>•Good weight values as compared to other bactoneurons (Table S4)<br><br>Selected as the final cellular device<br><br>Cellular device ANH2: Developed from ANH1 by changing the output from EGFP to tdTomato (Table S2) only for microscopic experiments.
BNeu 10	**Initial assumptions:** Based on the design knowledge of BNeu 2, BNeu 4 and BNeu 7, cellular device INH1 was designed (Figure S4 and table S2).<br><br>**Initial characterization of cellular device INH1: (Double-cassette system)** •Total leakage was low (Table S5)<br>•Difference between total leakage and highest leakage was Low (Table S5)<br>•Fold change between highest signal and highest leakage was more than 8 fold (Table S5)<br>•Good weight values as compared to other bactoneurons (Table S4)<br><br>Selected as the final cellular device<br><br>Cellular device INH2: Developed from INH1 by changing the output from EGFP to E2-Crimson (Table S2) only for microscopic experiments.

Table S8: Details of unit bactoneuron culturing. The optimized unit bactoneuron constructs are shown in bold.

**Unit bactoneuron**	**Cellular device**	**Transformation of plasmids**	**Overnight culture condition for transformed bacteria**	**Seeding of overnight uninduced culture**	**Induced culture condition**	**Time of propagation for induced culture for various experiments**				**Number of independent experiments performed in various days**
						**For fluorescence measurements with respect to the presence ('1' state) and absence ('0') of the input inducers in various combinations**	**For dose response experiments**	**For simulation validation experiments**	**For microscopic experiments**	
BNeu 1	IAA1	Chemically competent *E. coli* DH5αZ1 cells were transformed with plasmids corresponding to the cellular devices, recovered in fresh antibiotic-free LB media for 1 hour at 37 °C in shaking condition (~250 rpm), and grown for 12-15 hours in LB agar plates	We individually inoculated well-separated colonies from the transformed LB plates into fresh LB media supplemented with antibiotics without adding input inducers, and incubated for average 10 hours at 37 °C	The overnight uninduced single-colony LB culture was seeded into fresh LB media supplemented with appropriate antibiotics and inducers in the 100:1 ratio	37 °C at ~250 rpm in a bacterial shaking incubator	6 hours	12 hours	-	-	3
	IAA2					6 hours	12 hours	-	-	3
	IAA3					6 hours	12 hours	-	-	3
	IAA4					6 hours	12 hours	-	-	3
	IAA5					6 hours	12 hours	-	-	3
	IAA6					6 hours	12 hours	-	-	3
	IAA7A					12 hours	12 hours	-	-	3
	IAA8					12 hours	12 hours	-	-	3
	IAA9					12 hours	12 hours	-	-	3
	IAA10					12 hours	12 hours	-	-	3
	IAA11					12 hours	12 hours	-	-	3
	**IAA7B**					**10+6 hours**[a]	**10+6 hours**[a]	**10+6 hours**[a]	**10+6 hours**[a]	**5**
	IAA7B.A					-	-	-	10+6 hours[a]	1
	IAA7B.B					-	-	-	10+6 hours[a]	1
BNeu 2	INA1A					16 hours	-	-	-	1
	INA1B					16 hours	-	-	-	1
	INA1C					16 hours	-	-	-	1
	INA1D					16 hours	-	-	-	1
	INA2A					16 hours	-	-	-	1

	INA2B					16 hours	-	-	-	1
	INA2C					16 hours	-	-	-	1
	INA2D					16 hours	-	-	-	1
	INA3A					16 hours	-	-	-	1
	INA3B					16 hours	-	-	-	1
	INA3C					16 hours	-	-	-	1
	INA3D					16 hours	-	-	-	1
	INA4					16 hours	-	-	-	1
	INA5A					16 hours	-	-	-	1
	INA5B					16 hours	-	-	-	1
	INA5C					16 hours	-	-	-	1
	INA6A					16 hours	16 hours	-	-	3
	INA6B					16 hours	16 hours	-	-	3
	INA6C					16 hours	16 hours	-	-	3
	**INA7A**					**16 hours and 10+6 hours[a]**	**16 hours and 10+6 hours[a]**	**10+6 hours[a]**	**10+6 hours[a]**	**11**
	INA7B					16 hours	16 hours	-	-	3
	INA7C					16 hours	-	-	-	1
	INA7A.A					-	-	-	10+6 hours[a]	1
BNeu 3	**AAH1**					**10+6 hours[a]**	**10+6 hours[a]**	**10+6 hours[a]**	**10+6 hours[a]**	**6**
	AAH2					-	-	-	16 hours	1
BNeu 4	**IAH1**					**10+6 hours[a]**	**10+6 hours[a]**	**10+6 hours[a]**	**10+6 hours[a]**	**5**
	IAH2					-	-	-	16 hours	1
BNeu 5	**N1**					**10+6 hours[a]**	**10+6 hours[a]**	**10+6 hours[a]**	**-**	**4**
	N2					-	-	-	10+6 hours[a]	1
BNeu 6	ANI1A					16 hours	-	-	-	1
	ANI1B					16 hours	-	-	-	1
	ANI1C					16 hours	-	-	-	1
	ANI1D					16 hours	-	-	-	1
	ANI2A					16 hours	-	-	-	1
	ANI2B					16 hours	-	-	-	1
	**ANI2C**					**16 hours and 10+6 hours[a]**	**16 hours and 10+6 hours[a]**	**10+6 hours[a]**	**10+6 hours[a]**	**8**
	ANI2D					16 hours	-	-	-	1
	ANI3A					16 hours	-	-	-	1
	ANI3B					16 hours	-	-	-	1
	ANI3C					16 hours	-	-	-	1
	ANI3D					16 hours	-	-	-	1
	ANI4A					16 hours	-	-	-	1
	ANI4B					16 hours	-	-	-	1
	ANI4C					16 hours	-	-	-	1
	ANI4D					16 hours	-	-	-	1
	ANI2C.A					-	-	-	10+6 hours[a]	1
BNeu 7	**AHLB1**					**10+6 hours[a]**	**10+6 hours[a]**	**-**	**16 hours and 10+6 hours[a]**	**4**
	AHLB2					-	-	-	10+6 hours[a]	1
BNeu 8	**ATCB1**					**10+6 hours[a]**	**10+6 hours[a]**	**-**	**-**	**2**
	ATCB2					-	-	-	10+6 hours[a]	2
BNeu 9	**ANH1**					**10+6 hours[a]**	**10+6 hours[a]**	**10+6 hours[a]**	**-**	**4**
	ANH2					-	-	-	16 hours	1
BNeu 10	**INH1**					**10+6 hours[a]**	**10+6 hours[a]**	**10+6 hours[a]**	**-**	**4**
	INH2					-	-	-	16 hours	1

[a]10 hours induction of the 1% (V/V) overnight uninduced culture seeded into LB media with appropriate inducers and antibiotics followed by second seeding of 1% (V/V) of that induced culture into fresh media supplemented with inducers and antibiotics as appropriate and additional induction for 6 hours.

Table S9: List of bacterial strains and plasmids used in this study.

Plasmid name	Description	*Ori*	Antibiotic selection	Source
E. coli DH5α	-	-	-	Prof. David McMillen
E. coli DH5αZ1	-	-	-	Prof. David McMillen
pOR-EGFP-12	Source of EGFP gene and ColE1 *Ori*	ColE1	Amp	Prof. David McMillen
pOR-Luc-31	Source of p15A *Ori*	p15A	Cm	Prof. David McMillen
pmVenus-C1	Source of mVenus gene	pUC	Kan	Clontech
mTFP1-pBad (Plasmid#54553)	Source of mTFP1 gene	pBR322	Amp	Addgene

pUCP20T-E2Crimson (Plasmid#78473)	Source of E2-Crimson gene	pBR322	Amp	Addgene
mKO2-pBAD (Plasmid#54555)	Source of mKO2 gene	-	Amp	Addgene
pBW313lux-hrpR (Plasmid#61436)	Source of LuxR gene	p15A	Kan	Addgene
pXC3EGFP	EGFP gene with RBS R under P_{LUX}promoter: source of P_{LUX} promoter	p15A	Cm	This study
pTA1EGFP	EGFP gene with RBS R under $P_{LtetO-1}$ promoter	pUC	Amp	[1]
pTA2EGFP	EGFP gene with RBS R under $P_{LtetO-1}$ promoter	ColE1	Amp	[1]
pTA2E2-Crimson	E2-Crimson gene with RBS R under $P_{LtetO-1}$ promoter	ColE1	Amp	This study
pRC3EGFP	EGFP gene with RBS R under P_R promoter	p15A	Cm	[1]
pRC3MKO2	mKO2 gene with RBS R under P_R promoter	p15A	Cm	This study
pTA2cI	Source of wild type CI gene	ColE1	Amp	[1]
pRA1SEGFPTcIfm	Source of frame-shifted CI gene	pUC	Amp	[1]
pLA2ScIfmTcIfm	Frame-shifted CI gene with RBS R under both $P_{LlacO-1}$ promoter and $P_{LtetO-1}$ promoter	ColE1	Amp	[1]
pP_{IAA}1A1EGFP	EGFP gene with RBS R under P_{IAA}1promoter	pUC	Amp	This study
pP_{IAA}2A1EGFP	EGFP gene with RBS R under P_{IAA}2promoter	pUC	Amp	This study
pP_{IAA}3A1EGFP	EGFP gene with RBS R under P_{IAA}3promoter	pUC	Amp	This study
pP_{IAA}4A1EGFP	EGFP gene with RBS R under P_{IAA}4promoter	pUC	Amp	This study
pP_{IAA}5A1EGFP	EGFP gene with RBS R under P_{IAA}5promoter	pUC	Amp	This study
pP_{IAA}6A1EGFP	EGFP gene with RBS R under P_{IAA}6promoter	pUC	Amp	This study
pP_{IAA}7A1EGFP	EGFP gene with RBS R under P_{IAA}7promoter	pUC	Amp	This study
pP_{IAA}7C3EGFP	EGFP gene with RBS R under P_{IAA}7promoter	p15A	Cm	This study
pP_{IAA}7C3mVenus	mVenus gene with RBS R under P_{IAA}7promoter	p15A	Cm	This study
pP_{IAA}7C3E2-Crimson	E2-Crimson gene with RBS R under P_{IAA}7promoter	p15A	Cm	This study
pP_{IAA}8A1EGFP	EGFP gene with RBS R under P_{IAA}8promoter	pUC	Amp	This study
pP_{IAA}9A1EGFP	EGFP gene with RBS R under P_{IAA}9promoter	pUC	Amp	This study
pP_{IAA}10A1EGFP	EGFP gene with RBS R under P_{IAA}10promoter	pUC	Amp	This study
pP_{IAA}11A1EGFP	EGFP gene with RBS R under P_{IAA}11promoter	pUC	Amp	This study
pP_{INA}1A1EGFP	EGFP gene with RBS R under P_{INA}1promoter	pUC	Amp	This study
pP_{INA}2A1EGFP	EGFP gene with RBS R under P_{INA}2promoter	pUC	Amp	This study
pP_{INA}3A1EGFP	EGFP gene with RBS R under P_{INA}3promoter	pUC	Amp	This study
pP_{INA}4A1EGFP	EGFP gene with RBS R under P_{INA}4promoter	pUC	Amp	This study
pP_{INA}5A2EGFP(F)	EGFP gene with RBS R under P_{INA}5promoter (Forward direction)	ColE1	Amp	This study
pP_{INA}6A2EGFP(F)	EGFP gene with RBS R under P_{INA}6promoter (Forward direction)	ColE1	Amp	This study
pP_{INA}7A2EGFP(F)	EGFP gene with RBS R under P_{INA}7promoter (Forward direction)	ColE1	Amp	This study
pP_{INA}7A2mTFP1(F)	mTFP1 gene with RBS R under P_{INA}7promoter (Forward direction)	ColE1	Amp	This study
pP_{INA}7A2E2-Crimson(R)	E2-Crimson gene with RBS R under P_{INA}7promoter (Reverse direction)	ColE1	Amp	This study
pP_{ANI}1A2EGFP	EGFP gene with RBS R under P_{ANI}1promoter	ColE1	Amp	This study
pP_{ANI}2A2EGFP	EGFP gene with RBS R under P_{ANI}2promoter	ColE1	Amp	This study
pP_{ANI}2A2E2-Crimson	E2-Crimson gene with RBS R under P_{ANI}2promoter	ColE1	Amp	This study
pP_{ANI}2A2tdTomato(F)	tdTomato gene with RBS R under P_{ANI}2promoter (Forward direction)	ColE1	Amp	This study
pP_{ANI}3A2EGFP	EGFP gene with RBS R under P_{ANI}3promoter	ColE1	Amp	This study
pP_{ANI}4A2EGFP	EGFP gene with RBS R under P_{ANI}4promoter	ColE1	Amp	This study
pP_{AAH} C3EGFP(R)	EGFP gene with RBS R under P_{AAH} promoter (Reverse direction)	p15A	Cm	This study
pP_{AAH} C3RBSHEGFP(R)	EGFP gene with RBS RH under P_{AAH}promoter (Reverse direction)	p15A	Cm	This study
pP_{IAH} C3EGFP(R)	EGFP gene with RBS R under P_{IAH} promoter	p15A	Cm	This study
pTA2RBSC1EGFP	EGFP gene with RBS RC1 under $P_{LtetO-1}$ promoter	ColE1	Amp	This study
pTA2RBSC2EGFP	EGFP gene with RBS RC2 under $P_{LtetO-1}$ promoter	ColE1	Amp	This study
pTA2RBSC3EGFP	EGFP gene with RBS RC3 under $P_{LtetO-1}$ promoter	ColE1	Amp	This study
pTC3cI	Wild type CI gene with RBS R under $P_{LtetO-1}$ promoter	p15A	Cm	[1]
pTC3RBSC1cI	Wild type CI gene with RBS RC1 under $P_{LtetO-1}$ promoter	p15A	Cm	This study
pTC3RBSC2cI	Wild type CI gene with RBS RC2 under $P_{LtetO-1}$ promoter	p15A	Cm	This study
pTC3RBSC3cI	Wild type CI gene with RBS RC3 under $P_{LtetO-1}$ promoter	p15A	Cm	This study
pLC3cI	Wild type CI gene with RBS R under $P_{LlacO-1}$ promoter	p15A	Cm	This study
pLC3RBSC1cI	Wild type CI gene with RBS RC1 under $P_{LlacO-1}$ promoter	p15A	Cm	This study
pLC3RBSC2cI	Wild type CI gene with RBS RC2 under $P_{LlacO-1}$ promoter	p15A	Cm	This study
pLC3RBSC3cI	Wild type CI gene with RBS RC3 under $P_{LlacO-1}$ promoter	p15A	Cm	This study
pTA2LuxR	LuxR gene with RBS R under $P_{LtetO-1}$ promoter	ColE1	Amp	[2]

pJA2LuxR(F)	LuxR gene with RBS R under BBa_J23102 promoter (Forward direction)	ColE1	Amp	This study
pJA2LuxR(R)	LuxR gene with RBS R under BBa_J23102 promoter (Reverse direction)	ColE1	Amp	This study
pJA2LuxR(F)P_{INA}7E2-Crimson(R)	LuxR gene with RBS R under BBa_J23102 promoter (Forward direction) with E2-Crimson gene with RBS R under P_{INA}7 promoter (Reverse direction)	ColE1	Amp	This study
pP_{ANI}2A2tdTomato(F)JLuxR(R)	tdTomato gene with RBS R under P_{ANI}2promoter (Forward direction) with LuxR gene with RBS R under BBa_J23102 promoter (Reverse direction)	ColE1	Amp	This study
pXC3cIfm	Frame-shifted CI gene with RBS R under P_{LUX}promoter	p15A	Cm	This study
pX*C3EGFP	EGFP gene with RBS R under P_{LUX}^{*} promoter	p15A	Cm	This study
pX*C3E2-Crimson	E2-Crimson gene with RBS R under P_{LUX}^{*} promoter	p15A	Cm	This study
pA2MCS	Only MCS	ColE1	Amp	This study
pC3MCS	Only MCS	p15A	Cm	This study

Reference

1. K. Sarkar, S, Mukhopadhyay, D. Bonnerjee, R. Srivastava and S. Bagh, *J. Biol. Eng.,*2019, **13,** DOI: 10.1186/s13036-019-0151-x.
2. D. Bonnerjee, S. Mukhopadhyay, and S. Bagh, *Bioconjugate Chem.,* 2019, **30,** 3013-3020.
3. T. S. Moon, C. Lou, A. Tamsir, B. C. Stanton and C. A. Voigt, *Nature,* 2012, **491,** 249–253.
4. R. Lutz and H. Bujard, *Nucleic Acids Res.,* 1997, **25,** 1203–1210.
5. H. M. Salis, E. A. Mirsky and C. A. Voigt, *Nat. Biotechnol.,*2009, **27,** 946–950.
6. V. S. Mahajan, V. D. Marinescu, B. Chow, A. D. Wissner-Gross and P. Carr iGEM group: Antiquity, Data from “Registry of Standard Biological Parts”, 2003, http://parts.igem.org/Part:BBa_B0034.
7. K. F. Murphy, G. Bala´zsi and J. J. Collins, *Proc. Natl. Acad. Sci. U.S.A.,* 2007, **104,** 12726–12731.
